# Neural posterior estimation on exponential random graph models: evaluating bias and implementation challenges

**DOI:** 10.1007/s11222-026-10896-8

**Published:** 2026-05-17

**Authors:** Yefeng Fan, Simon Richard White

**Affiliations:** 1 MRC Biostatistics Unit, University of Cambridge, Cambridge, UK; 2 Department of Psychiatry, University of Cambridge, Cambridge, UK

**Keywords:** Bayesian ERGM, NPE, SNPE, Scalability

## Abstract

Exponential random graph models (ERGMs) are flexible probabilistic frameworks to model statistical networks through a variety of network summary statistics. Conventional Bayesian estimation for ERGMs involves iteratively exchanging with an auxiliary variable due to the intractability of the ERGM likelihood. However, this approach has limited scalability in large-scale implementations. Neural posterior estimation (NPE) is a recent advancement in simulation-based inference, using a neural network-based density estimator to infer the posterior for models with doubly intractable likelihoods for which simulations can be generated. While NPE has been successfully adopted in various fields such as cosmology, little research has investigated its use for ERGMs. Performing NPE on ERGMs not only provides a different approach to estimation for intractable ERGM likelihoods but also allows more efficient and scalable inference using the amortisation properties of NPE, and therefore we investigate how NPE can be effectively implemented in ERGMs. In this study, we present the first systematic implementation of NPE for ERGMs, rigorously evaluating potential biases, interpreting the bias magnitudes, and assessing computational costs. We compare NPE fits with conventional Bayesian ERGM fits as well as related neural simulation-based methods, namely neural likelihood estimation and neural ratio estimation. In our synthetic data analysis, we show that training a neural posterior estimator on 500,000 simulations circumvents the roughly 4,000,000,000 simulations required by conventional exchange-algorithm inference, enabling real-time posterior estimation. More importantly, our work highlights ERGM-specific areas that may pose particular challenges for the adoption of NPE.

## Introduction

Exponential random graph models (ERGMs) provide a general probabilistic framework to infer properties of statistical networks by considering a range of topological features through the incorporation of network summary statistics. ERGMs are highly flexible and can capture a wide range of network dependencies, and they have been applied to various fields including sociology and neuroimaging (Robins 2011; Harris 2013; Lusher et al. 2013; Lehmann et al. 2021). While past ERGM research was mainly performed under the frequentist setting with well-developed packages to facilitate its wider implementations (Hunter et al. 2008; Handcock et al. 2023; Krivitsky et al. 2023), the increasingly adopted Bayesian ERGMs allow fully probabilistic evaluation of uncertainty, inclusion of prior knowledge, and remedies to ERGM-specific degeneracy issues (Caimo and Friel 2011, 2017). Implementation of Bayesian ERGMs is subject to the double intractability issue, rooted in the intractable normalising constant in the ERGM likelihood due to the combinatorially large graph space. The most widely used approach to fitting Bayesian ERGMs is the exchange algorithm (Caimo and Friel 2011, 2017), using an auxiliary variable to explore the relation between the graph and parameter space and iteratively consider exchanging with the estimated parameters. It uses the augmented posterior trick to allow cancellation of the intractable normalising constants in the ERGM likelihood.

More generally, the exchange algorithm belongs to likelihood-free Bayesian inference or simulation-based inference. Approximate Bayesian computation (ABC) is a well-known likelihood-free approach used in past studies (Rubin 1984). More recently, a class of likelihood-free approaches called the neural posterior estimation (NPE) has taken on an important role in various studies (Papamakarios and Murray 2016; Greenberg et al. 2019; Papamakarios et al. 2019; Dax et al. 2021). NPE is a neural network-based approach to train a conditional estimator which models the Bayesian posterior distribution (Papamakarios and Murray 2016). In comparison with traditional ABC and the ERGM exchange algorithm, NPE can flexibly model high-dimensional parameter spaces using expressive neural network architectures such as normalising flows. In particular, NPE shows better amortisation, referring to minimal cost of re-fitting the model upon inference on a new observation, leading to more scalable inference (Avecilla et al. 2022; Pesonen et al. 2023). NPE has since been applied to a range of studies including cosmology and neuroscience (Gonçalves et al. 2020; Fengler et al. 2021; Vasist et al. 2023). Furthermore, several NPE variants have been proposed (Ward et al. 2022), and a well-rounded package has been developed (Tejero-Cantero et al. 2020). Among the NPE variants, a particularly important extension is the sequential neural posterior estimation (SNPE), which iteratively refines the conditional estimator towards the target posterior (Greenberg et al. 2019). Closely related neural simulation-based inference approaches include neural likelihood estimation (NLE) and neural ratio estimation (NRE) (Papamakarios et al. 2019; Hermans et al. 2020), which learn a surrogate likelihood or a likelihood-to-marginal ratio, respectively, and then typically rely on MCMC for posterior sampling.

In the past, alternative likelihood-free approximations have been performed on ERGMs. Examples are Kernel-based ABC and Copula ABC (Yin and Butts 2020; Karabatsos 2024). However, to our knowledge, NPE has not been performed on ERGMs. To date, the only related work is by Fen (2024), focusing on using SNPE to estimate general structural network models. While insightful, Fen (2024) does not address ERGMs directly, concluding that trouble arises in estimating ERGMs (without presenting any fitting results or discussing specific details). Another very recent and partially related work is performed by Mele (2025), focusing on the frequentist setting rather than the Bayesian setting. Mele (2025) proposes training a feed-forward neural network to infer the parameters. This approach functions similarly to the general NPE literature but with a different objective function, contributing as an alternative to the conventional Markov Chain Monte Carlo maximum likelihood estimation (MCMCMLE) used in frequentist ERGM estimation (Hunter and Handcock 2006; Hunter et al. 2008; Hummel et al. 2012). While important to the development of ERGMs, their work fundamentally differs from our study in terms of both research interest and inference paradigm.

Therefore, this study fills several important gaps in the existing literature. Section 2 introduces the methodological background on ERGMs, NPE, and SNPE. Section 3 then presents a simulation study that rigorously and systematically evaluates the overall suitability of NPE for ERGMs. Using synthetic network data, we assess potential sources of bias, quantify their magnitudes by mapping results back to the graph (summary statistics) space, and compare the fitting performance of NPE with conventional Bayesian ERGM methods. We also examine the computational cost structure of NPE relative to Bayesian ERGM fitting in order to clarify the settings in which amortisation can yield practical efficiency gains. Section 4 demonstrates these neural approaches on a real-world network and provides a comparative evaluation of NPE against two closely related neural methods, namely NLE and NRE. Across both the simulation and empirical studies, we show that NPE can be a suitable approach for the ERGM settings, while also identifying ERGM-specific features that are particularly likely to pose challenges for NPE and SNPE implementations. These issues are discussed systematically in Section 5. Overall, this study establishes a strong foundation for NPE-based inference for ERGMs and provides practical guidance for diagnosing fitting issues in future implementations.

## Methodology

### Exponential random graph model

ERGMs characterise the probability distribution of a random network $\textbf{Y}$ with observed realisation $\textbf{y}$, such that the topological structure of the observed network $\textbf{y}$ can be explained by a set of summary statistics (sufficient statistics) $\textbf{h}(\textbf{y})\in \mathbb {R}^{p}$, which can flexibly include a wide range of summary statistics and nodal covariates (Harris 2013; Lusher et al. 2013).

$$\begin{aligned} \textbf{Y} \sim \text {ERGM}(\boldsymbol{\theta }) \quad p(\textbf{Y}=\textbf{y}\mid \boldsymbol{\theta })=\dfrac{\exp \!\bigl (\boldsymbol{\theta }^{\mathsf T}\textbf{h}(\textbf{y})\bigr )}{c(\boldsymbol{\theta })}, \end{aligned}$$

where $\boldsymbol{\theta } \in \mathbb {R}^{p}$ is a vector of parameters associated with the summary statistics, and $c(\boldsymbol{\theta })$ is the normalising constant of the form,

$$\begin{aligned} c(\boldsymbol{\theta })=\sum _{\textbf{y}'\in \mathcal {Y}} \exp \!\bigl (\boldsymbol{\theta }^{\mathsf T}\textbf{h}(\textbf{y}')\bigr ). \end{aligned}$$

The normalising constant is typically intractable due to the combinatorially large graph space $\mathcal {Y}$, and this makes the sampling distribution analytically intractable, which forms the main difficulty in fitting an ERGM.

In the Bayesian setup, a common ERGM estimation procedure is the approximate exchange algorithm (Caimo and Friel 2011, 2017). It iteratively explores the mapping between the parameter space and the graph space by introducing an auxiliary variable to get around the doubly intractable problem, allowing cancellation of the intractable normalising constants. More specifically, given a prior distribution $\pi (\boldsymbol{\theta })$, the algorithm instead targets an augmented posterior

$$\begin{aligned}\pi (\boldsymbol{\theta },\boldsymbol{\theta }',\textbf{y}' \mid \textbf{y}) \propto \pi (\boldsymbol{\theta })\,p(\textbf{y}\mid \boldsymbol{\theta })\, g(\boldsymbol{\theta }'\mid \boldsymbol{\theta })\,p(\textbf{y}'\mid \boldsymbol{\theta }'),\end{aligned}$$

whose marginal in θ is the original posterior $\pi (\boldsymbol{\theta }\mid \textbf{y})$. The auxiliary variable $\textbf{y}' \sim p(\cdot \mid \boldsymbol{\theta }')$ is simulated with the ERGM density characterised by $\boldsymbol{\theta }'$, where the simulation algorithm is given in Appendix A. We use a symmetric normal proposal $g(\boldsymbol{\theta }'\mid \boldsymbol{\theta })=\mathcal {N}(\boldsymbol{\theta }, \mathbf {\Sigma })$ with proposal covariance matrix $\mathbf {\Sigma }$. We can accept $\boldsymbol{\theta }'$ with the acceptance probability

1 $$\begin{aligned} \begin{aligned} \text {AR}&= \frac{\pi (\boldsymbol{\theta }' \mid \textbf{y})}{\pi (\boldsymbol{\theta } \mid \textbf{y})} \cdot \frac{p(\textbf{y}' \mid \boldsymbol{\theta })}{p(\textbf{y}' \mid \boldsymbol{\theta }')}\\&= \frac{\exp \!\bigl (\boldsymbol{\theta }'^{\mathsf T}\textbf{h}(\textbf{y})\bigr )\,\pi (\boldsymbol{\theta }')\,c(\boldsymbol{\theta })\; \exp \!\bigl (\boldsymbol{\theta }^{\mathsf T}\textbf{h}(\textbf{y}')\bigr )\,c(\boldsymbol{\theta }')}{\exp \!\bigl (\boldsymbol{\theta }^{\mathsf T}\textbf{h}(\textbf{y})\bigr )\,\pi (\boldsymbol{\theta })\,c(\boldsymbol{\theta }')\; \exp \!\bigl (\boldsymbol{\theta }'^{\mathsf T}\textbf{h}(\textbf{y}')\bigr )\,c(\boldsymbol{\theta })}\\&= \frac{\pi (\boldsymbol{\theta }')}{\pi (\boldsymbol{\theta })}\, \exp \!\Bigl \{ \bigl (\boldsymbol{\theta }'-\boldsymbol{\theta }\bigr )^{\mathsf T}\textbf{h}(\textbf{y}) + \bigl (\boldsymbol{\theta }-\boldsymbol{\theta }'\bigr )^{\mathsf T}\textbf{h}(\textbf{y}') \Bigr \}. \end{aligned} \end{aligned}$$

The estimation procedure is summarised in Algorithm 1.

**Algorithm 1:**
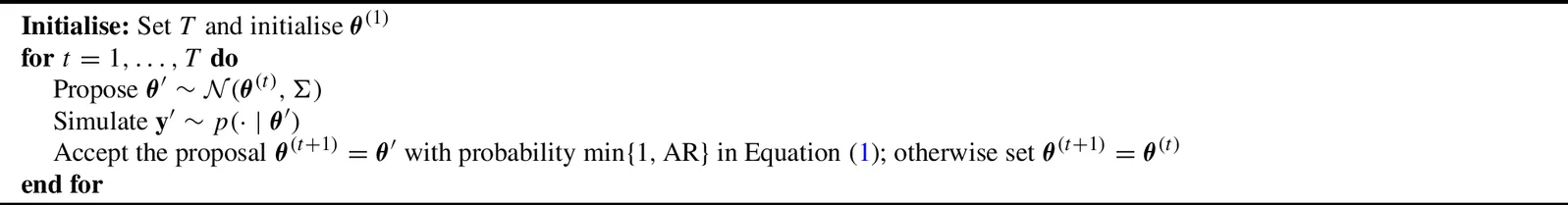
Approximate Exchange Algorithm

#### Summary statistics and network sizes

In our analysis we consider the following summary statistics: the number of edges, geometrically weighted edgewise shared partners (GWESP), and geometrically weighted non-edgewise shared partners (GWNSP), which are defined as:

$$\begin{aligned} &  h_{GWESP}(\textbf{y}) = \exp (\tau )\sum _{i=1}^{n-2}\left( 1 - \left( 1 - \exp (-\tau )\right) ^{i}\right) p_{i},\\ &  h_{GWNSP}(\textbf{y}) = \exp (\tau )\sum _{i=1}^{n-2}\left( 1 - \left( 1 - \exp (-\tau )\right) ^{i}\right) np_{i}, \end{aligned}$$

where *n* is the number of vertices. $p_{i}$ and $np_{i}$ are the number of connected and non-connected vertex pairs sharing *i* neighbours, respectively.

Summary statistics selections, and their impact on NPE, are not the focus of this paper; we choose this combination of summary statistics based on the fact that it has been successfully adopted in different past studies and has important inference implications on network efficiencies (Simpson et al. 2011; Lehmann et al. 2021), and we set the decay parameter τ=0.75 as per previous studies (Lehmann et al. 2021).

Furthermore, we consider 90-vertex networks in this study, which aligns with the past neuro-imaging ERGM implementations on the real-world data from the Cambridge Centre for Ageing and Neuroscience project (Shafto et al. 2014; Lehmann et al. 2021). Networks of this form ensure our findings can be generalised to moderate-sized real-world network applications.

### Neural posterior estimation

#### Data definition and sufficiency

Before introducing NPE, we clarify the observed data used for inference. Specifically, we treat the summary statistics $\textbf{h}(\textbf{y})$ as the observed data rather than the network $\textbf{y}$ itself. This is because it is challenging for neural networks to model high-dimensional graph outputs (Labbé et al. 2009), and graph isomorphism (the same graph can be labelled differently) further adds to the complexity (McKay and Piperno 2014).

We can model the relationship between model parameters and summary statistics $\textbf{h}(\textbf{y})$ instead of modelling between parameters and realised graph $\textbf{y}$ using the Bayes sufficiency of $\textbf{h}(\textbf{y})$ for parameter $\boldsymbol{\theta }$ (see Appendix B),

$$\begin{aligned} \pi (\boldsymbol{\theta }\mid \textbf{y})=\pi \left( \boldsymbol{\theta }\mid \textbf{h}\left( \textbf{y}\right) \right) . \end{aligned}$$

Therefore, for inference on $\boldsymbol{\theta }$ we do not need additional information from the network $\textbf{y}$. To be clear in the following sections, we use $\textbf{x}$ to denote the statistics, such that $\textbf{x}=\textbf{h}(\textbf{y})$, and $\textbf{x}_{\text {obs}}$ represents the observed network statistics.

#### Amortised neural posterior estimation

NPE was first proposed by Papamakarios and Murray (2016) and targets learning a parametric approximation to the exact posterior distribution $\pi (\boldsymbol{\theta }\mid \textbf{x})$ using a neural network.

In the first simulation stage, a set of *B* data-parameter pairs $\{\boldsymbol{\theta }_{b}, \textbf{x}_{b}\}_{b=1}^{B}$ are simulated, forming our training dataset. The parameters may be simulated from any proposal distribution $\tilde{p}(\boldsymbol{\theta })$, but it is often chosen to be the prior (i.e., $\tilde{p}(\boldsymbol{\theta })=\pi (\boldsymbol{\theta })$),

$$\begin{aligned} &  \boldsymbol{\theta }_{b} \sim \tilde{p}(\boldsymbol{\theta }),\\ &  \textbf{x}_{b} \sim p(\textbf{x}\mid \boldsymbol{\theta }_{b}).\end{aligned}$$

The simulation pairs are used to train a conditional density estimator denoted as $q_{\phi }(\boldsymbol{\theta }\mid \textbf{x})$, where ϕ parametrises the inference neural network. In this paper, we mainly use the masked autoregressive flow (MAF) as the conditional density estimator (Papamakarios et al. 2017), and we summarise alternative conditional density estimators and their usage in Appendix C as this is not the focus of our study.

In the next training step, the conditional density estimator is trained through ϕ by maximising the likelihood of the data-parameter pairs. More specifically, the objective function is,

$$\begin{aligned} \mathcal {L}(\phi ) = -\mathbb {E}_{\boldsymbol{\theta }, \textbf{x}} [ \log q_\phi (\boldsymbol{\theta } \mid \textbf{x}) ],\end{aligned}$$

where the expectation is taken with respect to $p(\boldsymbol{\theta }, \textbf{x})=p(\textbf{x}\mid \boldsymbol{\theta })\tilde{p}(\boldsymbol{\theta })$. The objective function can be approximated using the training data such that we minimise the negative log-likelihood,

$$\begin{aligned} \mathcal {L}(\phi ) \approx -\sum _{b=1}^{B}\log q_{\phi }(\boldsymbol{\theta }_{b}\mid \textbf{x}_{b}).\end{aligned}$$

Papamakarios and Murray (2016) proved that as $B \rightarrow \infty$, the training density estimator becomes,

2 $$\begin{aligned} q_{\phi }(\boldsymbol{\theta }\mid \textbf{x}) \propto \dfrac{\tilde{p}(\boldsymbol{\theta })}{\pi (\boldsymbol{\theta })}\pi (\boldsymbol{\theta }\mid \textbf{x}). \end{aligned}$$

If we simulate directly from the prior distribution (i.e., $\tilde{p}(\boldsymbol{\theta })=\pi (\boldsymbol{\theta })$), the trained density is a direct estimator of the posterior distribution. For the implementation of amortised NPE, we consider only simulating from the prior distribution (i.e., setting $\tilde{p}(\boldsymbol{\theta })=\pi (\boldsymbol{\theta })$).

In the final inference step, the trained conditional density estimator that targets the posterior distribution is conditioned on a particular observation $\textbf{x}_{\text {obs}}$ such that the parameter inference is performed on $q_{\phi }(\boldsymbol{\theta }\mid \textbf{x}_{\text {obs}})$. The trained density estimator is amortised, meaning inference can be performed on different observations without the need to re-fit the model.

#### Sequential neural posterior estimation

The amortisation advantage of NPE is only useful if the trained conditional density estimator is sufficiently accurate, which depends on having sufficient data-parameter pairs within the target posterior parameter domain.

To address insufficient coverage of training pairs in the high-posterior-density region, we consider SNPE, which iteratively refines the proposal to concentrate simulations near the target posterior. However, Equation (2) implies that when the proposal $\tilde{p}(\boldsymbol{\theta })$ differs from the prior $\pi (\boldsymbol{\theta })$, the density estimator trained instead targets the proposal posterior $\tilde{p}(\boldsymbol{\theta }\mid \textbf{x})$ rather than $\pi (\boldsymbol{\theta }\mid \textbf{x})$, namely

3 $$\begin{aligned} \tilde{p}(\boldsymbol{\theta }\mid \textbf{x}) = \pi (\boldsymbol{\theta }\mid \textbf{x}) \dfrac{\tilde{p}(\boldsymbol{\theta })p(\textbf{x})}{\pi (\boldsymbol{\theta })\tilde{p}(\textbf{x})}, \end{aligned}$$

where

$$\begin{aligned} \tilde{p}(\textbf{x})=\int \tilde{p}(\boldsymbol{\theta })p(\textbf{x}\mid \boldsymbol{\theta })\,d\boldsymbol{\theta }.\end{aligned}$$

Following Greenberg et al. (2019), we parameterise an estimator of the true posterior by $q_{\phi }(\boldsymbol{\theta }\mid \textbf{x})$, and define a transformed density $\tilde{q}_{\phi }(\boldsymbol{\theta }\mid \textbf{x})$ which corresponds to the proposal posterior when simulations are generated from $\tilde{p}(\boldsymbol{\theta })$,

4 $$\begin{aligned} \tilde{q}_{\phi }(\boldsymbol{\theta }\mid \textbf{x})=q_{\phi }(\boldsymbol{\theta }\mid \textbf{x})\dfrac{\tilde{p}(\boldsymbol{\theta })}{\pi (\boldsymbol{\theta })}\dfrac{1}{Z_{\phi }(\textbf{x})}. \end{aligned}$$

$Z_{\phi }(\textbf{x})$ is the normalising constant,

$$\begin{aligned} Z_{\phi }(\textbf{x})=\int q_{\phi }(\boldsymbol{\theta }\mid \textbf{x})\dfrac{\tilde{p}(\boldsymbol{\theta })}{\pi (\boldsymbol{\theta })}\,d\boldsymbol{\theta }.\end{aligned}$$

This format aligns with Equation (3), because when $q_{\phi }(\boldsymbol{\theta }\mid \textbf{x})=\pi (\boldsymbol{\theta }\mid \textbf{x})$, we have $Z_{\phi }(\textbf{x})=\tilde{p}(\textbf{x})/p(\textbf{x})$ and hence $\tilde{q}_{\phi }(\boldsymbol{\theta }\mid \textbf{x})=\tilde{p}(\boldsymbol{\theta }\mid \textbf{x})$. Therefore, by training the transformed density $\tilde{q}_{\phi }(\boldsymbol{\theta }\mid \textbf{x})$ using proposal simulations, we can directly“read off” $q_{\phi }(\boldsymbol{\theta }\mid \textbf{x})$ as an estimator of the target posterior without post-processing.

However, the integral $Z_{\phi }(\textbf{x})$ cannot always be evaluated. This depends on the choice of the proposal distribution and the type of conditional density estimator. MAF is a case where $Z_{\phi }(\textbf{x})$ is not analytically evaluatable. To address this issue, Greenberg et al. (2019) used an atomic loss to flexibly incorporate various proposal distributions and density estimators, for example MAF. However, this flexibility comes with the potential risk of so-called leakage issues, where the estimated posterior distribution produced by a neural network assigns substantial probability mass to regions of the parameter space with very low probability under the true posterior distribution. We summarise the atomic objective in Appendix D and discuss leakage in our implementation results.

When training SNPE across rounds, we minimise

$$\begin{aligned} \mathcal {L}(\phi )=-\sum _{b=1}^{B} \log \tilde{q}_{\phi }(\boldsymbol{\theta }_{b}\mid \textbf{x}_{b}).\end{aligned}$$

Using the proposition of Greenberg et al. (2019) (Equation (2)), as $B \rightarrow \infty$, $\tilde{q}_{\phi }(\boldsymbol{\theta }\mid \textbf{x}) \rightarrow \tilde{p}(\boldsymbol{\theta }\mid \textbf{x})$ and $q_{\phi }(\boldsymbol{\theta }\mid \textbf{x}) \rightarrow \pi (\boldsymbol{\theta }\mid \textbf{x})$.

SNPE iteratively refines the proposal by starting from $\tilde{p}_1(\boldsymbol{\theta })=\pi (\boldsymbol{\theta })$ and then setting subsequent proposals using the current posterior estimate $q_{\phi }(\boldsymbol{\theta }\mid \textbf{x}_{\text {obs}})$, re-using simulations from previous rounds. Due to this iterative refinement, the amortisation property is lost, hence SNPE must be retrained for every new observation. We outline SNPE in Algorithm 2.

**Algorithm 2:**
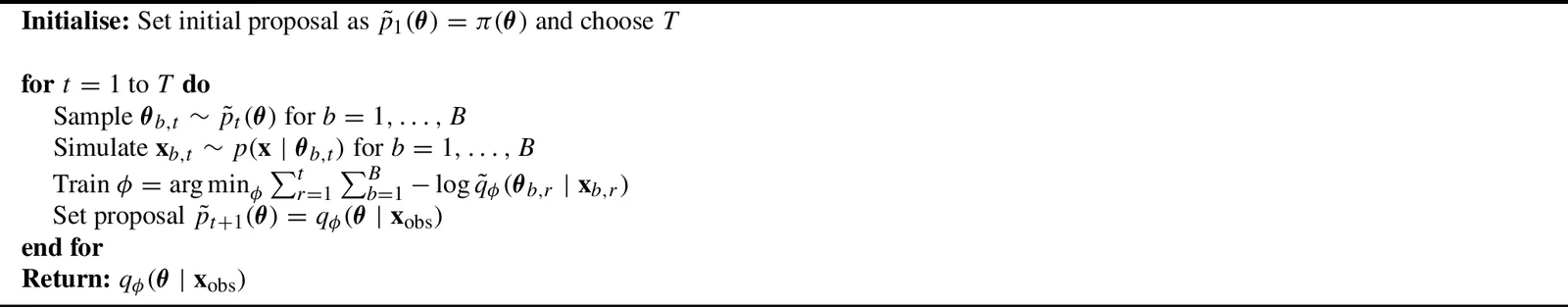
Sequential Neural Posterior Estimation (SNPE)

## Simulation study

### Setup

We have four aims in our exploration of the application of NPE to ERGMs. First, we assess whether NPE is appropriate for ERGMs by evaluating bias and quantifying its magnitude. Second, we identify settings in which NPE performs poorly, investigate likely causes, and summarise them as cautionary scenarios. Third, we compare NPE with standard Bayesian ERGM fits. Fourth, we assess computational cost and practical efficiency via a dedicated cost study.

For SNPE, we examine how iterative refinement improves targeting of the posterior for an ERGM. We also study the role of coverage, focusing on behaviour when the initial prior or proposal places insufficient mass near the“true”posterior region, which in other applications can lead to leakage (Tejero-Cantero et al. 2020; Deistler et al. 2022). We outline each implementation setup in the subsections below.

#### Bias evaluation design

To evaluate the bias of NPE, we use synthetic data, so the “true”parameter is known. Let $\boldsymbol{\theta }_{\text {true}}^{k}$, k=1,⋯,K, denote a set of *K*“true”parameters. For each $\boldsymbol{\theta }_{\text {true}}^{k}$, we simulate *M* independent summary statistics as observations $\textbf{x}_{m}^{k}$, m=1,⋯,M. We then evaluate the amortised NPE posterior for each $\textbf{x}_{m}^{k}$, compute the posterior mean $\hat{\boldsymbol{\theta }}_{m}^{k}$, and average over *m* to obtain a point estimate for bias quantification

$$\begin{aligned} \hat{\boldsymbol{\theta }}^{k}=\frac{1}{M}\sum _{m=1}^{M}\hat{\boldsymbol{\theta }}_{m}^{k}. \end{aligned}$$

Based on computational constraints, we set K=40. More specifically, to avoid collinearity issues for geometrically weighted statistics (Hunter 2007; Hunter et al. 2008), we restrict our attention to the sparse graph space with less than 1,100 edges (approximated via exploratory simulations across $\boldsymbol{\theta }$ for 90-vertex networks, which indicated strong overlap and collinearity in the geometrically weighted statistics beyond 1,100 edges). Therefore, we sample 10 parameter sets in each edge-count stratum: [0, 275), [275, 550), [550, 825), and [825, 1, 100).

Averaging over *M* reduces the impact of stochastic variability under the ERGM, since the mapping between $\boldsymbol{\theta }$ and $\textbf{x}$ is not one-to-one and may be multi-modal (i.e., a single realisation is not representative of its corresponding $\boldsymbol{\theta }_{\text {true}}$). Using Wilcoxon rank-sum comparisons across candidate *M*, we set $M=1{,}000$. Overall, this yields 40, 000 NPE fits, and for each fit, we draw 100, 000 posterior samples.

We use three error summaries to assess estimation bias:

1. Mean Error (ME):
  $$\begin{aligned} \textbf{ME} = \frac{1}{K} \sum _{k=1}^{K} \left( \hat{\boldsymbol{\theta }}^{k}-\boldsymbol{\theta }_{\text {true}}^{k}\right) .\end{aligned}$$
2. Mean Absolute Error (MAE):
  $$\begin{aligned} \textbf{MAE} = \frac{1}{K} \sum _{k=1}^{K} \left| \hat{\boldsymbol{\theta }}^{k}-\boldsymbol{\theta }_{\text {true}}^{k}\right| .\end{aligned}$$
3. Root Mean Square Error (RMSE):
  $$\begin{aligned}\textbf{RMSE} = \sqrt{\frac{1}{K} \sum _{k=1}^{K} \left( \hat{\boldsymbol{\theta }}^{k}-\boldsymbol{\theta }_{\text {true}}^{k}\right) \odot \left( \hat{\boldsymbol{\theta }}^{k}-\boldsymbol{\theta }_{\text {true}}^{k}\right) }.\end{aligned}$$

For NPE, we use a MAF with 50 hidden units and 5 transformations (package default setup; see Appendix C). For amortised NPE (and for Bayesian ERGM comparisons), we use the prior $\boldsymbol{\theta }\sim \mathcal {N}(\textbf{0},10\textbf{I})$, where $\textbf{I}$ is the 3×3 identity matrix. We simulate $B=500{,}000$ training pairs from the prior.

#### Bias magnitude design

We then aim to quantify the magnitude of the assessed NPE biases. Because discrepancies in ERGM parameter space can be difficult to interpret, we map bias back to the graph space (summary-statistics space).

More specifically, for each $\boldsymbol{\theta }_{\text {true}}^{k}$, we generate reference network samples representing the “true”data distribution,

$$\begin{aligned} \textbf{x}^{k}_{\text {true}} = \{ \textbf{x}_{\text {true}, 1}^{k}, \dots , \textbf{x}_{\text {true}, 100{,}000}^{k} \}. \end{aligned}$$

We compare this with posterior predictive samples based on the NPE fits. Specifically, for each $m=1,\dots ,1{,}000$ we simulate from the ERGM at the posterior mean $\hat{\boldsymbol{\theta }}_{m}^{k}$ to account for inherent ERGM variability, generating 100 realisations per *m* (therefore, 100,000 in total),

$$\begin{aligned} \textbf{x}^{k} = \bigcup _{m=1}^{1{,}000} \{ \textbf{x}_{m, 1}^{k}, \dots , \textbf{x}_{m, 100}^{k} \}. \end{aligned}$$

Our main interest is coverage rather than the full distributional shape. We classify bias as small if, for each statistic component, the mean of $\textbf{x}^{k}$ lies within the 5th and 95th percentiles of $\textbf{x}^{k}_{\text {true}}$. We also report the empirical coverage rate (overlap between $\textbf{x}^{k}_{\text {true}}$ and $\textbf{x}^{k}$, relative to $\textbf{x}^{k}_{\text {true}}$) for cases classified as having large bias.

#### Bayesian ERGM comparison design

To compare NPE with standard Bayesian ERGM, we use the bergm package (5.0.7). We set a burn-in period of 1, 000 iterations for each fit and generate 6, 000 posterior samples. The Bayesian ERGM uses the same prior distribution as the amortised NPE for consistency.

Similar to the NPE analysis, we account for inherent ERGM variance by repeatedly fitting the Bayesian ERGM on 1, 000 realisations, and we compute and compare the posterior means from each fit.

The computational demands of fitting Bayesian ERGMs are significantly higher than those of NPE due to the lack of amortisation. This makes it infeasible to explore all forty $\boldsymbol{\theta }_{\text {true}}$ values or to increase the number of iterations and posterior samples. Therefore, we consider four $\boldsymbol{\theta }_{\text {true}}$ cases for comparison (resulting in a total of 4, 000 Bayesian ERGM fits). We choose two $\boldsymbol{\theta }_{\text {true}}$ cases where NPE shows small to moderate bias and two cases where NPE exhibits significantly large bias (Table 1). In particular, Case 3 corresponds to the $\boldsymbol{\theta }_{\text {true}}$ value with the highest NPE bias among all 40 tested cases.

**Table 1:** Selected $\boldsymbol{\theta }_{\text {true}}$ values and evaluated NPE bias using absolute error. Each number corresponds, in order, to the statistics: number of edges, GWESP, and GWNSP; four cases are considered; Case 1 and Case 2 represent small to moderate NPE bias; Case 3 and Case 4 represent large NPE bias; $\textbf{AE} =\left| \hat{\boldsymbol{\theta }}^{k}-\boldsymbol{\theta }_{\text {true}}^{k}\right| .$

	**Case 1**			**Case 2**		
	Edges	GWESP	GWNSP	Edges	GWESP	GWNSP
$\boldsymbol{\theta }_{\text {true}}$	0.37	-0.28	6.79	-1.54	-1.01	0.72
AE	0.20	0.03	0.09	0.03	0.01	0.01
	**Case 3**			**Case 4**		
	Edges	GWESP	GWNSP	Edges	GWESP	GWNSP
$\boldsymbol{\theta }_{\text {true}}$	-11.96	0.27	1.05	-5.79	1.13	0.61
AE	5.19	0.15	0.41	1.01	0.34	0.09

#### Cost assessment design

NPE shows efficiency gains through amortisation and is especially useful in large-sample analysis. We rigorously assess the cost of NPE and compare it with standard Bayesian ERGMs. Furthermore, although hyperparameter tuning of the neural network structure is not our research focus and we use the default setup in the sbi (version 0.24) package, we also assess how architecture choices affect NPE performance and cost. For this assessment, we use 90-node networks and the prior $\mathcal {N}(\textbf{0},10\textbf{I})$. GPU computations are performed on an NVIDIA GeForce RTX 5060 Laptop GPU (7.96 GB, compute capability 12.0) using CUDA version 12.6. CPU-based computations are on an AMD Ryzen AI 9 HX 370 processor with Radeon 890M graphics (base frequency 2.00 GHz).

To assess the fitting cost for the standard Bayesian ERGMs, we first consider network simulation cost by repeating 1, 000 independent ERGM simulations for each auxiliary MCMC length (4, 000, 10, 000 and 50, 000) and recording wall-clock time. We then record full Bayesian ERGM fitting time with bergm (version 5.0.7; Algorithm 1) with burn-in 1, 000 and 100, 000 posterior samples. We repeat and average fitting times across five independent estimations under each auxiliary MCMC length.

For NPE assessment, we first simulate 100, 000 data-parameter pairs from the prior under 10, 000 auxiliary network simulation iterations. We then consider four combinations of training data size *B* and flow architecture (number of hidden units *H* and the number of flow transformations *L*):

1. $$B = 50{,}000$$, H=50, L=10,
2. $$B = 50{,}000$$, H=100, L=10,
3. $$B = 50{,}000$$, H=50, L=20,
4. $$B = 100{,}000$$, H=50, L=10.

For each configuration, we use a training dataset of size *B* by drawing without replacement from the 100, 000 data-parameter pairs and train an NPE with a MAF flow using the chosen *H* and *L*. This is repeated 5 times per configuration with random initialisations.

To assess accuracy, we use the Case 1 parameters in Section 3.1.3 ($\boldsymbol{\theta } = (0.37,\,-0.28,\,6.79)$) and evaluate bias with 1, 000 simulations as observed and 100, 000 posterior samples for each observed similar to Section 3.1.1 scheme.

#### SNPE design

We use SNPE to examine how iterative refinement improves posterior targeting for ERGMs, and to assess sensitivity to prior (proposal) coverage.

**Fig. 1:**
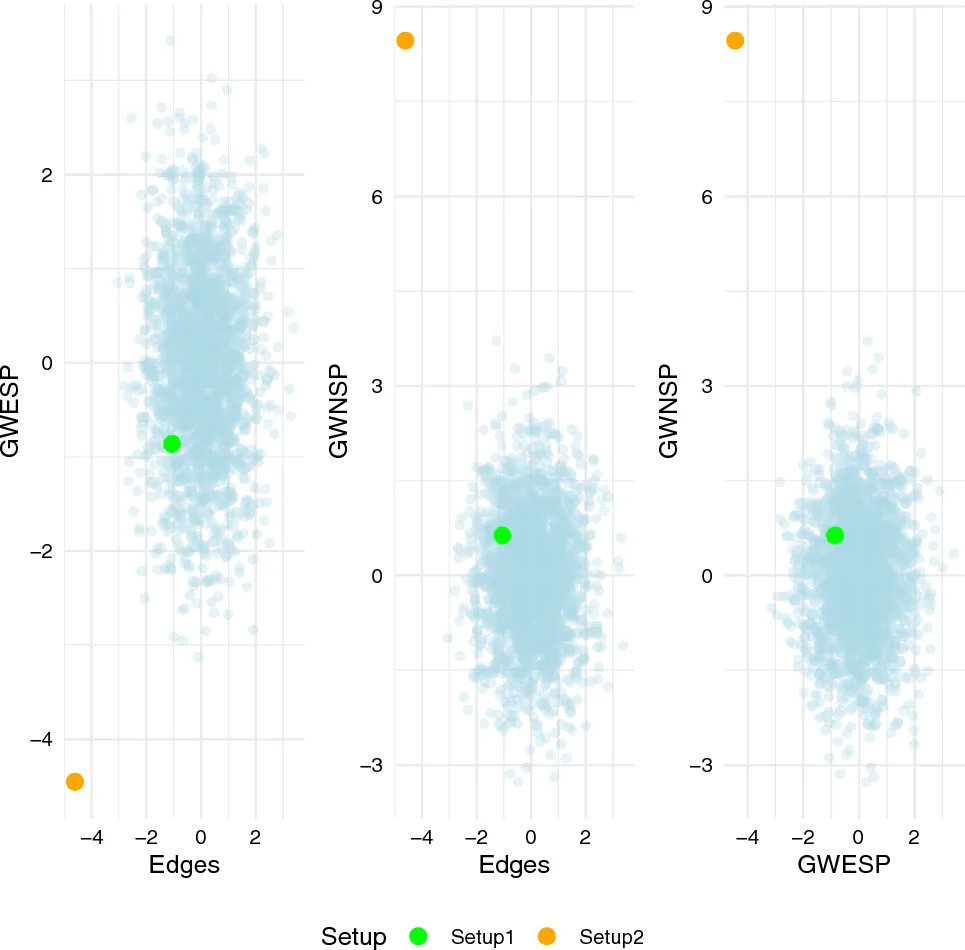
Illustration of selected $\boldsymbol{\theta }_{\text {true}}$ cases to implement SNPE; Setup 1 is the green point, and the parameters are: -1.05, -0.86, and 0.63; Setup 2 is the orange point, and the parameters are: -4.61, -4.45, and 8.46; the domain of blue points represents the coverage provided by the prior.

We run a simulation study with two“true”parameter settings $\boldsymbol{\theta }_{\text {true}}$ (Figure 1). Because SNPE is not amortised and targets a particular observation, we restrict attention to these two cases and use a more constrained prior (hence initial proposal) $\pi (\boldsymbol{\theta })\sim \mathcal {N}(\textbf{0},\textbf{I})$.

In Setup 1, we choose $\boldsymbol{\theta }_{\text {true}}$ within the prior support and perform $B=100{,}000$ simulations per round for 5 SNPE rounds (total 500, 000 data-parameter pairs). We also perform a reduced setting with $B=1{,}000$ per round for 8 rounds to study behaviour under crude initial estimates.

In Setup 2, we choose $\boldsymbol{\theta }_{\text {true}}$ outside the prior coverage to study the impact of inadequate coverage, and the remaining SNPE settings match Setup 1.

Across all SNPE experiments, we use the same MAF architecture as in the amortised NPE.

### Bias evaluation results

We fit an amortised NPE as described in the implementation scheme, and the evaluated biases are presented in Table 2 (A) using three metrics.

Although absolute biases in parameter space can be difficult to interpret directly, the assessed NPE biases are generally small. This conclusion is NPE-specific because averaging over repeated ERGM realisations reduces the impact of ERGM stochastic variability.

ME and MAE differ substantially in scale, suggesting that signed biases often cancel in ME. RMSE penalises extreme errors more heavily, and correspondingly shows larger errors. While most cases exhibit small biases, we identify twelve $\boldsymbol{\theta }_{\text {true}}$ cases where the absolute bias exceeds 1.5 for at least one parameter; seven of these twelve cases exhibit multimodal behaviour.

NPE also performs relatively poorly in the edge-count stratum [0, 275). Seven of the ten cases in this stratum correspond to graph spaces near the empty graph, highlighting“boundary effects”, which we discuss in Section 5.

### Bias magnitude results

We project the NPE results to the data space (graph space) to yield more interpretable bias measurements and assess posterior predictive fit.

Using the pre-designed significance criteria (Section 3.1.2), the majority of cases are accurate under NPE (Table 2 (B)). Discrepancies for GWESP are more severe than for the other two statistics, which may reflect greater sensitivity of GWESP in sparse graphs.

We examine cases classified as having large bias under the summary statistics criterion. Table 2 (C) reports coverage percentages comparing $\textbf{x}^{k}$ with $\textbf{x}^{k}_{\text {true}}$. For most large-bias cases, NPE still shows moderate overall posterior predictive coverage, typically with one statistic exhibiting poorer coverage. Particularly, in five of the eight large-bias cases, the observed summary statistics generated at the“true”parameter lie near the boundary of the statistic space (often close to zero), again highlighting boundary effects.

Overall, quantifying bias in both parameter space and summary-statistics space indicates that NPE performs well in most cases.

**Table 2:** Table summarising the evaluated NPE biases and their bias magnitudes quantification; (A) Evaluated NPE biases summarised using three metrics for all 40 cases: ME, MAE, and RMSE; (B) The percentages of cases that are regarded as insignificant bias using our quantification criteria in the data space; (C) Posterior predictive coverage percentages for the summary statistics in cases classified as having large discrepancy.

	**Edges**	**GWESP**	**GWNSP**
**A. Bias Metrics**			
ME	-0.01	-0.30	-0.09
MAE	0.61	0.61	0.38
RMSE	1.09	1.04	0.82
**B. Insignificant Bias Percentages**			
Insignificant bias rate	92.5%	82.5%	95.0%
**Case (k)**	**Edges**	**GWESP**	**GWNSP**
**C. Large-bias Cases Predictive Coverage**			
**2**	60.31%	0.02%	71.93%
**5**	79.36%	0.68%	81.43%
**8**	3.71%	65.33%	100.00%
**9**	45.67%	38.17%	47.92%
**10**	34.58%	29.58%	36.32%
**11**	58.79%	31.47%	64.64%
**13**	39.57%	39.88%	23.55%
**20**	84.42%	13.37%	79.65%

### Bayesian ERGM comparison results

We compare the NPE fits with the Bayesian ERGM fits, and as explained in Section 3.1.3, we consider four cases as outlined in Table 1.

**Fig. 2:**
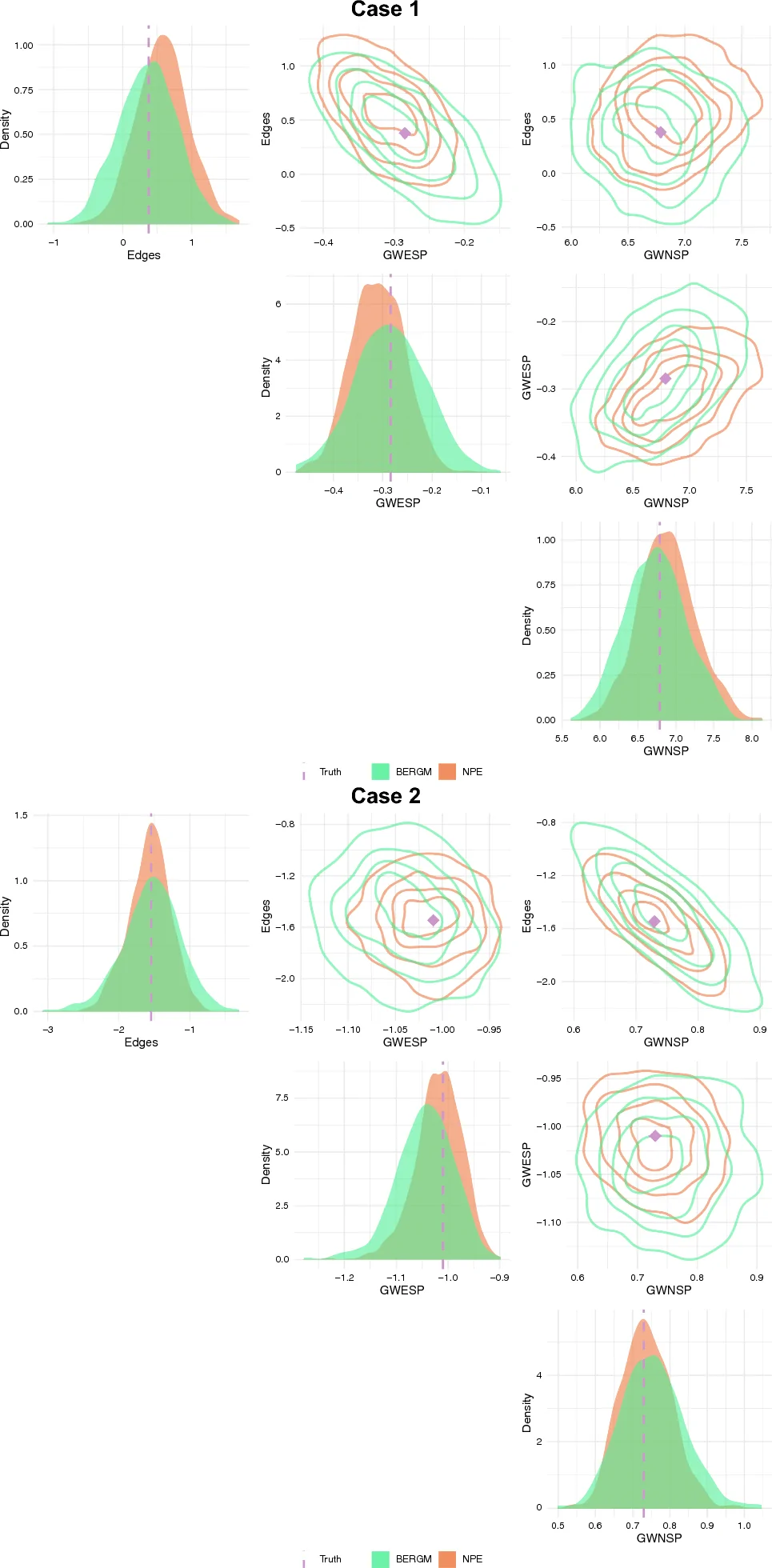
Plots for comparing NPE and Bayesian ERGM for Case 1 and Case 2; posterior density plots for parameters edges, GWESP and GWNSP (diagonal); Contour plots for pairwise posterior density spaces (off-diagonal); plotted for posterior means for NPE (orange) and Bayesian ERGM (green); the corresponding“truth”is plotted in purple (dashed lines and points).

In Cases 1 and 2 (Figure 2), NPE and Bayesian ERGM yield similar posterior distributions, centred near the “true” parameter values. NPE also captures posterior correlations between parameters (e.g., between edges and GWNSP in Case 2), consistent with Bayesian ERGM. In these cases, we find that NPE performs comparably to Bayesian ERGM.

**Fig. 3:**
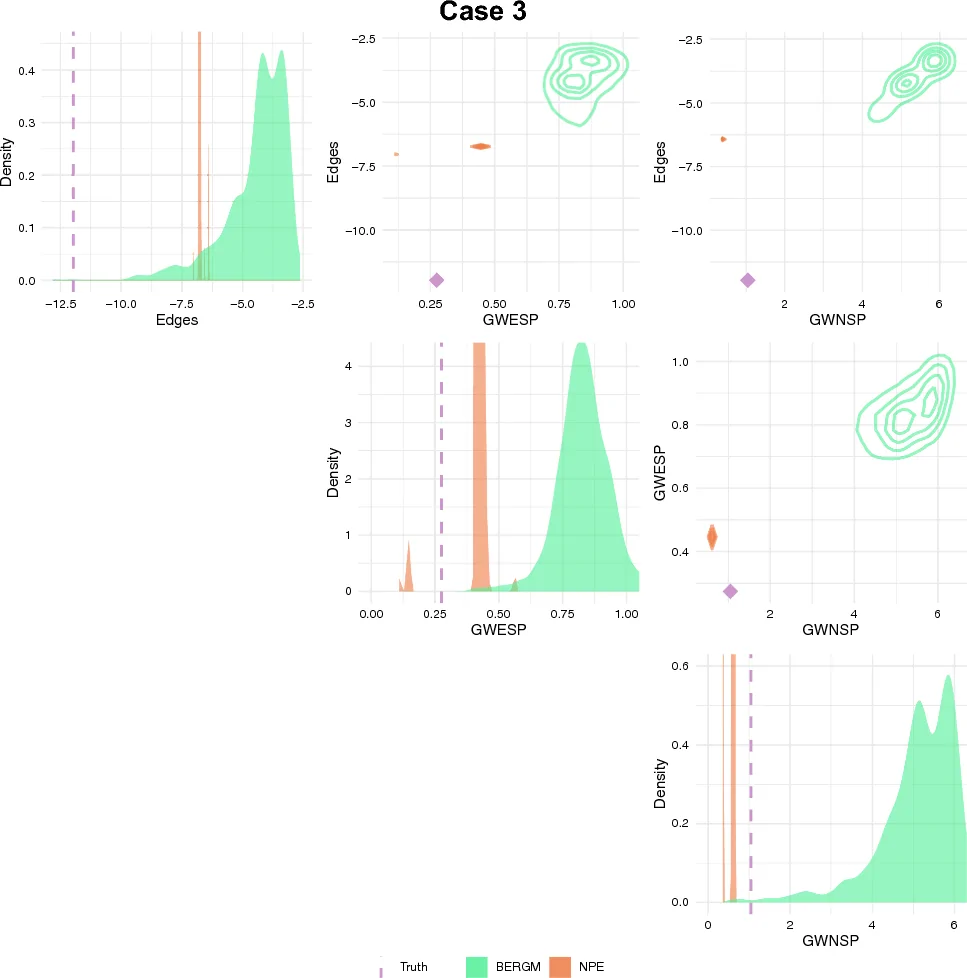
Plots for comparing NPE and Bayesian ERGM for Case 3; posterior density plots for parameters edges, GWESP and GWNSP (diagonal); Contour plots for pairwise posterior density spaces (off-diagonal); plotted for posterior means for NPE (orange) and Bayesian ERGM (green); the corresponding“truth”is plotted in purple (dashed lines and points).

Case 3 corresponds to a $\boldsymbol{\theta }_{\text {true}}$ that yields statistics near the empty graph and produces the largest NPE bias among the 40 tested cases. As shown in Figure 3, Bayesian ERGM also performs poorly in recovering $\boldsymbol{\theta }_{\text {true}}$, with substantial deviation from the “true” parameter. This illustrates boundary effects, where a large region of parameter space can map to near-extreme graphs, making inference difficult.

**Fig. 4:**
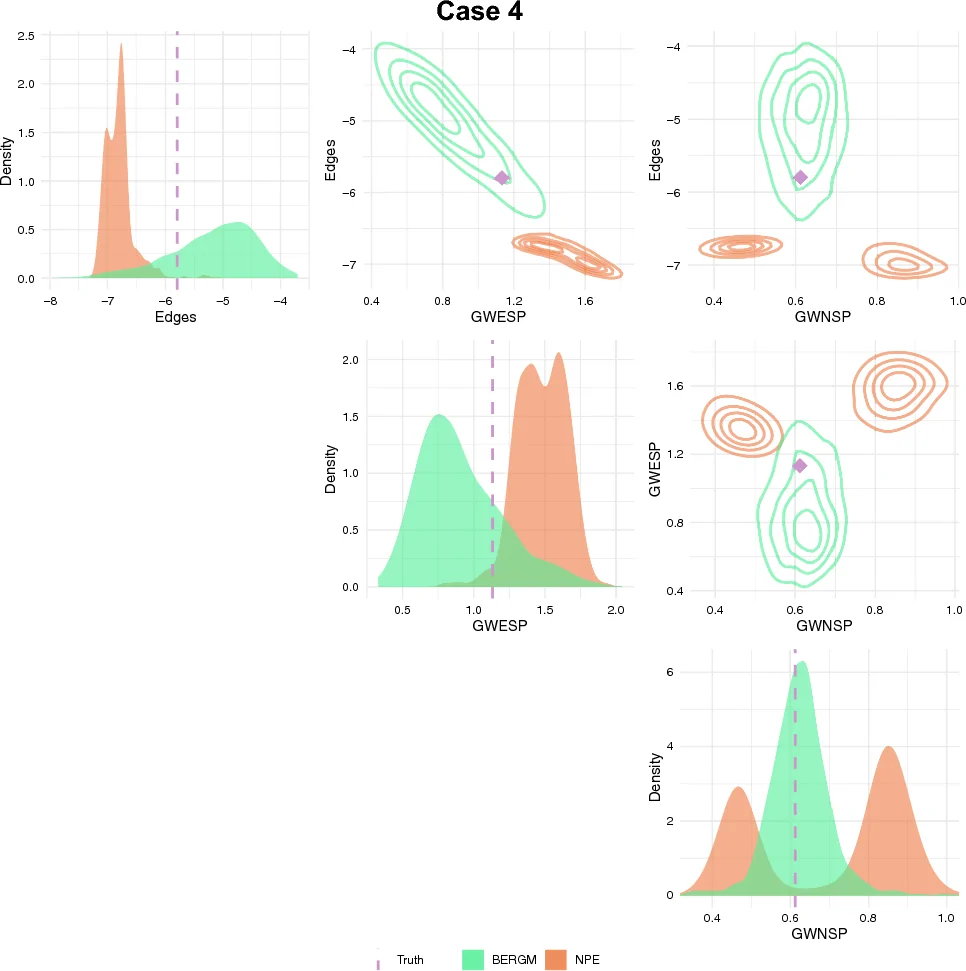
Plots for comparing NPE and Bayesian ERGM for Case 4; posterior density plots for parameters edges, GWESP and GWNSP (diagonal); Contour plots for pairwise posterior density spaces (off-diagonal); plotted for posterior means for NPE (orange) and Bayesian ERGM (green); the corresponding“truth”is plotted in purple (dashed lines and points); **Explanation**: Case 4 is a bimodal case, especially in GWNSP. Both the NPE and Bayesian ERGM perform poorly. Case 4 shows bimodality when initialising at the full network and using 50,000 network simulation iterations. Adjusting the simulation setup may change the distribution, which will be discussed later.

In Case 4 (Figure 4), we observe bimodality in the data space, mainly in GWNSP, and NPE shows two clusters of posterior mass. Both Bayesian ERGM and NPE perform poorly, with posterior (means) densities showing substantial deviation from the“true”parameter.

Overall, we have shown that NPE can achieve the same level of accuracy as the Bayesian ERGM. However, we should be more cautious in cases that are naturally difficult to estimate.

### Cost assessment results

We analyse the fitting cost structure of a standard Bayesian ERGM and our NPE approach, and discuss their scaling properties. We then approximate the break-even point in terms of the number of posterior samples *S* and the number of networks *J* to show the efficiency gain of NPE.

#### Cost model for standard bayesian ERGM

For a standard Bayesian ERGM fitted by the exchange algorithm (Algorithm 1), the dominant cost is repeated auxiliary network simulation. Let $I_{\textrm{aux}}$ be the number of auxiliary iterations per MCMC step and let $C_1(I_{\textrm{aux}})$ be the cost of simulating one network using $I_{\textrm{aux}}$ iterations. The total cost is

5 $$\begin{aligned} C_{\textrm{BERGM}} \approx c\, S\, C_1(I_{\textrm{aux}}), \end{aligned}$$

where $C_1(I_{\textrm{aux}})$ grows approximately linearly in $I_{\textrm{aux}}$ (Table 3) and increases rapidly with network size (Lusher et al. 2013; Tolochko and Boomgaarden 2024). In implementations such as Bergm, batching, vectorised linear algebra, and other overhead reductions are absorbed into a dimensionless factor c>0, which we estimate as c≈0.33, 0.48, and 0.88 for $I_{\textrm{aux}}=4{,}000$, 10, 000, and 50, 000, respectively (Table 3 and Table 4). As $I_{\textrm{aux}}$ increases, simulation dominates runtime.

#### Cost model for NPE

For NPE, the total fitting cost decomposes into simulation, training, and inference,

6 $$\begin{aligned} C_{\textrm{NPE}} = B \, C_1(I_{\textrm{aux}}) + C_2(B, H, L) + J \, S \, C_3(H, L), \end{aligned}$$

where *B* is the number of simulated training pairs, *H* is the number of hidden units in the flow, and *L* is the number of flow transformations.

The simulation cost $B\,C_1(I_{\textrm{aux}})$ grows linearly in *B* and inherits the dependence of $C_1$ on $I_{\textrm{aux}}$ and network size. This term is shared with the standard Bayesian ERGM. A larger *B* improves coverage and reduces Monte Carlo noise in the NPE training.

$C_2(B, H, L)$ is the training cost. Larger *B* leads to more gradient updates per epoch and therefore longer training time. Increasing the number of transformations *L* deepens the flow and increases expressiveness, but replicates the neural blocks and increases cost more sharply. Changing *H* modifies the width of each block. The timings in Table 5 show training time increasing with *B* and *L*, while *H* exhibits a small and somewhat counterintuitive decreasing effect, likely reflecting optimisation variability.

$J S C_3(H,L)$ is the inference cost of drawing *S* posterior samples for each of *J* networks. Once the flow is fitted, each draw is obtained by a forward pass (amortisation) with cost that depends only on (*H*, *L*). For *J* networks and *S* draws per network, the sampling cost scales linearly in *J* and *S*, but with a very small constant. In Table 5, the measured inference times correspond to 100, 000, 000 draws, which makes $J S C_3(H,L)$ negligible compared with other cost components. As a result, once the NPE has been trained, the marginal cost of adding more networks for inference is minor.

Finally, Table 5 shows little change in posterior bias summaries across configurations despite noticeable differences in training time, suggesting diminishing returns once *B* provides adequate coverage and the flow is sufficiently expressive. In our setting, residual bias is dominated by the quality and noise of the simulated training data rather than limited flow expressiveness.

**Table 3:** Table of average wall-clock time for a single network ERGM auxiliary simulation; times are based on the average over 1, 000 repetitions under each setting.

$I_{\textrm{aux}}$	mean (s)	sd (s)
4,000	0.03308	0.01098
10,000	0.05163	0.01538
50,000	0.13654	0.02156

**Table 4:** Table of average wall-clock time for fitting a standard Bayesian ERGM to a single observed network; with burn-in 1, 000 and 100, 000 posterior draws; times are averaged over 5 repetitions per setting.

$I_{\textrm{aux}}$	mean (s)	sd (s)
4,000	1,118.2	33.18
10,000	2,520.8	56.31
50,000	12,068.7	948.60

#### Cost equating and break-even point

To compare the two approaches, we equate the total cost of fitting on *J* networks with *S* posterior draws per network under BERGM (Equation 5) and NPE (Equation 6),

$$\begin{aligned} &  \begin{aligned} J \, c \, S \, C_1(I_{\textrm{aux}})&= B \, C_1(I_{\textrm{aux}}) + C_2(B,H,L) \\&\quad + J \, S \, C_3(H,L). \end{aligned} \\ &  J= \frac{B \, C_1(I_{\textrm{aux}}) + C_2(B,H,L)}{S \, \bigl (c \, C_1(I_{\textrm{aux}}) - C_3(H,L)\bigr )}.\end{aligned}$$

Plugging in our assessed empirical timings at $I_{\textrm{aux}}=10{,}000$ (Tables 3 to 5), we find that for $S \approx 10^5$ posterior draws per network, the break-even occurs at only a small number of networks (around one to three, depending on the chosen *B* and flow architecture). Beyond this point, the NPE becomes strictly more efficient than refitting a full BERGM for each additional network, and the efficiency gap widens linearly as *J* increases.

**Table 5:** Table of average NPE training time and amortised inference time, and MAE, computed within each repetition as the mean absolute error across 1,000 evaluation networks, then averaged over 5 repetitions; build time denotes the one-time cost of preparing the trained posterior for sampling; inference time is reported for 100, 000, 000 posterior samples (1,000 evaluation networks and 100,000 posteriors per network; Section 3.1.4); wall-clock times are averaged over 5 repetitions per configuration.

*H*	*L*	*B*	Train (s)	Build (s)	Infer (s)	MAE edges	MAE GWESP	MAE GWNSP
50	10	50,000	1,311.3	0.045	183.0	0.366	0.067	0.310
100	10	50,000	1,115.4	0.039	185.0	0.376	0.066	0.327
50	20	50,000	2,020.6	0.068	343.2	0.420	0.067	0.436
50	10	100,000	2,391.9	0.088	175.3	0.395	0.090	0.602

### SNPE results

#### Setup 1

**Fig. 5:**
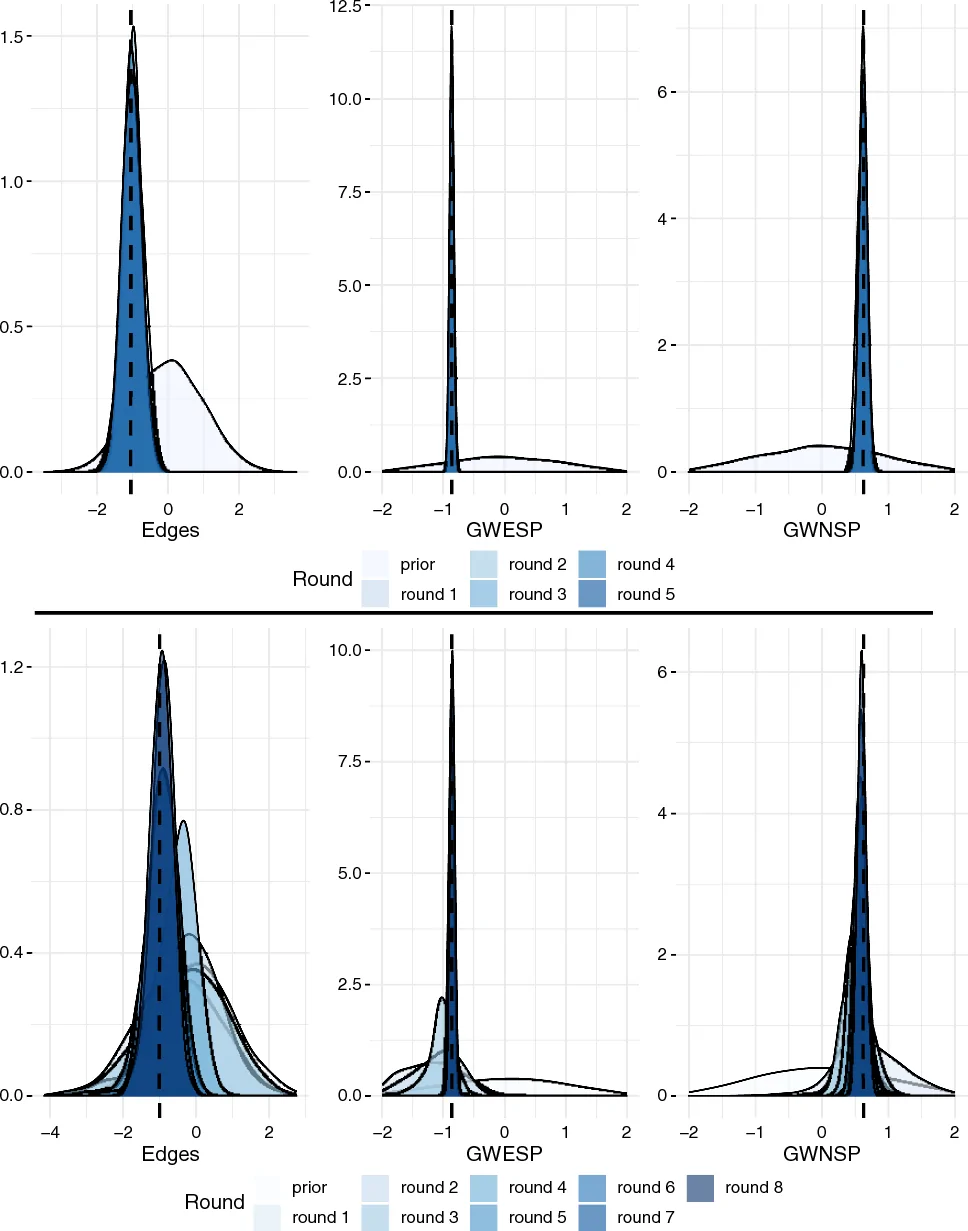
Density plots for posterior densities across SNPE rounds; later rounds are presented with lower transparencies and deeper colours; the plots are for the SNPE Setup 1 (top) and Setup 1 reduced (bottom); the Setup 1 shows 5 rounds; the Setup 1 reduced shows 8 rounds; each density plot considers 100,000 posterior samples; the“true”parameters are plotted as the black dashed lines

In Setup 1, the target observation corresponds to“true” parameter values that lie within the coverage of the prior distribution (Figure 1). In Figure 5 (top), with $B=100{,}000$, the posterior densities stabilise after Round 1 (Round 1 is equivalent to amortised NPE), with little additional refinement in later rounds. This suggests that first-round simulations from the prior already provide adequate information for posterior inference when the target lies well within the prior coverage.

We therefore consider a more challenging case with a smaller per-round simulation budget. In Figure 5 (bottom), we observe progressive improvement across SNPE rounds. The posterior mass concentrates and posterior variance decreases. This illustrates how SNPE can refine the posterior through iterative proposal updates, often achieving accurate inference with fewer total simulations than amortised NPE (Greenberg et al. 2019). In Setup 1 reduced, the posterior appears to stabilise after 8 rounds using 8, 000 simulated pairs in total.

#### Setup 2

**Fig. 6:**
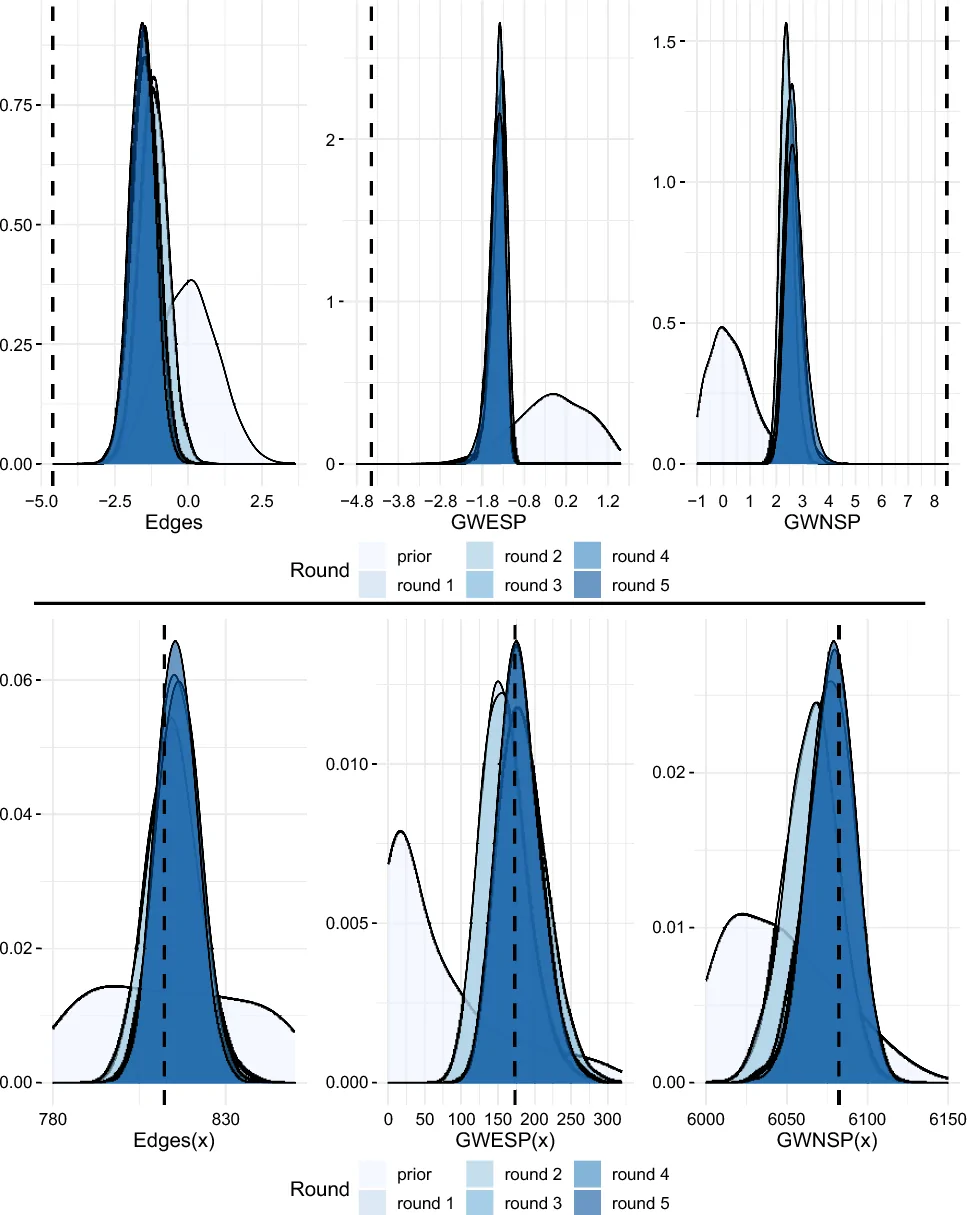
Density plots for posterior densities across 5 SNPE rounds for the Setup 2 (top); density plots for posterior predictive across 5 SNPE rounds (bottom); later rounds are presented with lower transparencies and deeper colours; each density plot considers 100,000 posterior samples and 5,000 posterior predictive samples; the “true” parameters and observed statistics are plotted as the black dashed lines.

In Setup 2, we consider a case where the coverage is not sufficiently wide to provide support for the “true” posterior. In Figure 6 (top), SNPE does not recover the “true” parameter, although the estimates move in the“correct”directions of summary statistics tendencies. This is because SNPE uses atomic loss to return a distribution that is only proportional to the true posterior in support of the proposal.

In other applications, insufficient coverage may also lead to leakage (Tejero-Cantero et al. 2020; Deistler et al. 2022). In our ERGM experiments, we did not observe severe leakage under poor coverage. By examining posterior predictive simulations, we find that the posterior predictive distribution remains relatively close to the observed statistics (Figure 6 bottom). A likely explanation is that dependence among the summary statistics permits alternative parameter values that match key statistics reasonably well, even when the parameters are far from the “true” values. This highlights the risk of inadequate proposal support. In ERGM implementations, without knowing the “true” parameter, inadequate coverage may be masked by seemingly adequate posterior predictive checks. This coverage issue also has further implications for ERGM implementations, which we discuss later.

## Lazega lawyers friendship network example

### Data description and implementation

We present a real-data implementation using the Lazega lawyers friendship network (Lazega 2001), both as a more realistic test case for NPE and as a setting to compare NPE with two related neural simulation-based approaches, NLE and NRE (see Appendix E). We also use a standard Bayesian ERGM as a baseline.

Starting from the directed friendship relation, we construct an undirected network $\textbf{y}_{\text {obs}}$ on 71 vertices, retaining all actors and placing an edge whenever either lawyer reports the other as a friend. Summary statistics (edges, GWESP, and GWNSP) are used for this implementation, consistent with our previous analysis. The observed network has 399 edges, and GWESP and GWNSP equal 753.74 and 1769.31, respectively.

The prior on the ERGM parameters is $\boldsymbol{\theta }\sim \mathcal {N}(\textbf{0},9\textbf{I})$, chosen to offer a plausible coverage for the posterior.

For all three neural methods, we simulate shared data-parameter pairs as a training dataset from the prior of size $B = 100{,}000$. For consistency, all network simulation procedures, including those used in Bayesian ERGM fitting, share the same setup, for which we use the observed network as the initial network and set the auxiliary MCMC length to 2, 485 (full network size of the observed network).

For consistent and comparable implementations, fitted NPE and NLE share the same MAF structure (50 hidden units and 5 transformations), used as the conditional density estimator for NPE and conditional likelihood model for NLE, matching the setup in Section 3.1.1. For NRE, we use a naive classifier $D_{\varphi }(\boldsymbol{\theta },\textbf{x})$, implemented as a multi-layer perceptron (MLP) with 50 hidden units. Full implementation code is provided in the Supplementary Material.

We generate 100, 000 posterior samples from all fitted models and analyse both the posterior distributions and the posterior predictive distributions, with plots based on a subsample of 10, 000 draws for computational efficiency.

### Results

**Fig. 7:**
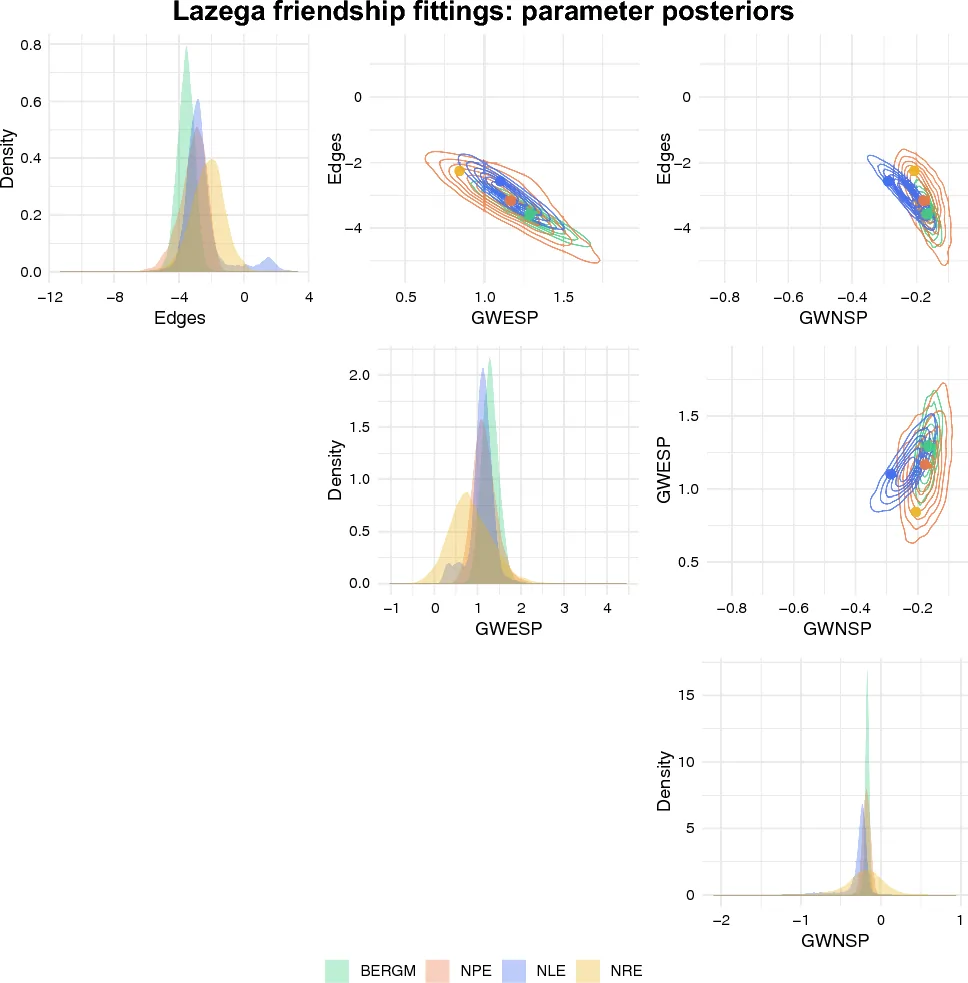
Plots comparing the standard Bayesian ERGM, NPE, NLE and NRE, implemented on the Lazega friendship network; posterior density plots for parameters: edges, GWESP and GWNSP (diagonal); contour plots for pairwise posterior density spaces (off-diagonal); some multi-modal contours, especially for NRE, are largely neglected because they are too wide and distort the plot.

**Fig. 8:**
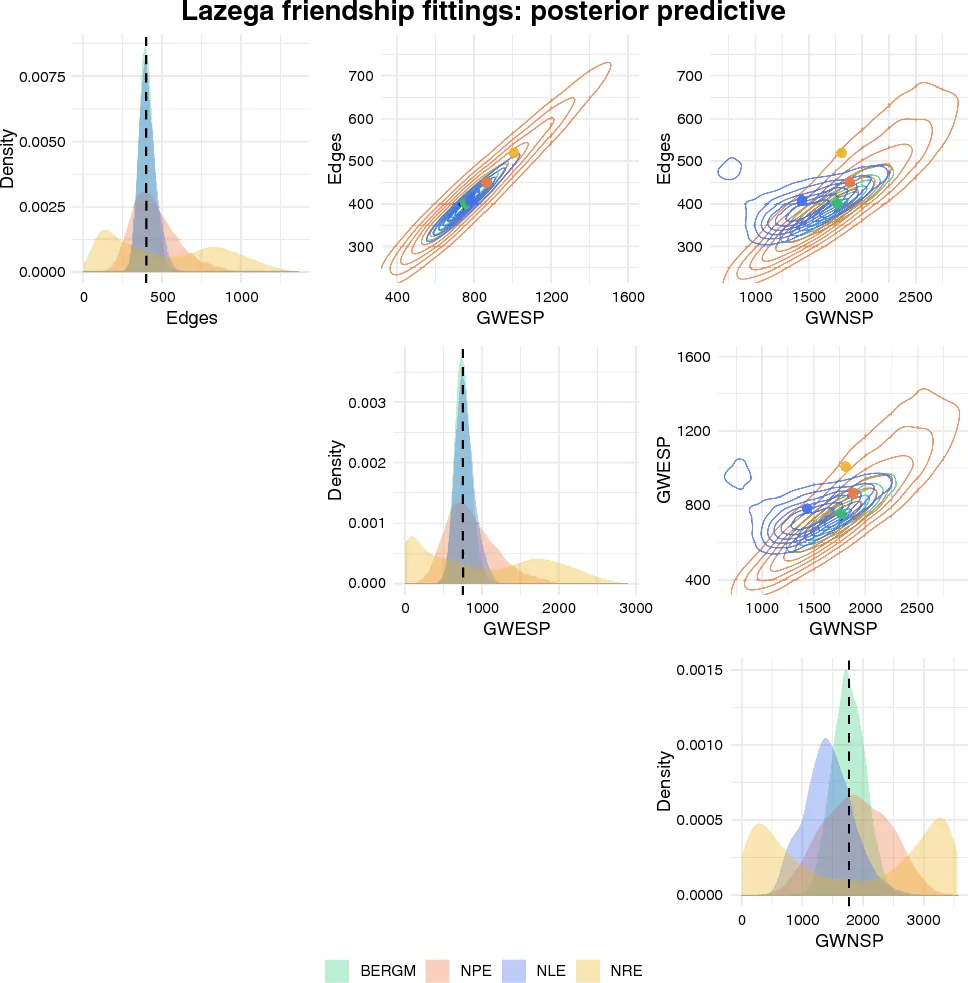
Plots comparing the standard Bayesian ERGM, NPE, NLE and NRE, implemented on the Lazega friendship network; posterior predictive density plots for edges, GWESP and GWNSP (diagonal); contour plots for pairwise posterior predictive spaces (off-diagonal); some multi-modal contours, especially for NRE, are largely neglected because they are too wide and distort the plot; the observed statistics are plotted as black dashed lines.

For the posterior (Figure 7), NPE yields posterior means closest to the Bayesian ERGM, and its contours largely cover the Bayesian ERGM posterior, but the distribution is more dispersed across all three parameters. NLE is also broadly consistent with the Bayesian ERGM, with partial overlap, but exhibits bimodality and deviations in GWNSP. NRE shows the weakest agreement, with a substantially wider spread. For the posterior predictive (Figure 8), NPE reproduces the observed statistics reasonably well and is close to the Bayesian ERGM in predictive means, but remains over-dispersed. NLE aligns well with the Bayesian ERGM for edges and GWESP, but shows systematic deviation in GWNSP and isolated regions of predictive density due to its bimodal posterior. NRE produces a misaligned, bimodal predictive distribution.

For NPE, we observe a well-centred but more dispersed posterior and posterior predictive, both broadly consistent with the Bayesian ERGM reference. Such over-dispersion is not specific to ERGM applications and has been reported for prior-based, single-round NPE (Duan et al. 2025; Macias et al. 2025). The training objective behaves like a mass-covering divergence, penalising the estimator more for missing probability mass where the target posterior has support than for assigning some extra mass around it. In practice, this may be improved by using SNPE or more expressive flow architectures.

For NLE, we may perceive it as training an efficient network sampler. NLE learns a fast surrogate for the likelihood $p(\textbf{x}\mid \boldsymbol{\theta })$, based on our simulated training dataset. After training, inference remains conceptually close to the standard Bayesian ERGM in that MCMC is still required. This retains MCMC per observed network and therefore loses posterior level amortisation, and miscalibration in the surrogate may cause deviations (here, most noticeably in GWNSP in Figure 8).

For NRE, the cross-entropy objective prioritises classification between joint and product samples over the global prior-predictive space, rather than accurate recovery of the likelihood ratio in the narrow region where the posterior concentrates. As a result, a classifier can perform well overall while being poorly calibrated on a high-density ridge around the posterior, and small miscalibrations can translate into large multiplicative errors in the posterior density. In the ERGM setting, the non-linear mapping from parameters to network statistics may further induce spurious modes or artificial tail behaviour, causing the bimodality and poor fit we observe. Our use of a relatively simple MLP classifier may also limit ratio calibration, making NRE more fragile than NLE and NPE in this implementation.

We next compare training and inference costs (Table 6). NPE is amortised at the posterior level and avoids post-training MCMC, so posterior sampling for any observed network (with the same size and summary statistics) is extremely fast (0.18s for $10^5$ posterior samples in our implementation). NLE and NRE are not amortised at the posterior level and still require MCMC per observed network. They reduce the need for ERGM simulator calls by using learned likelihood (NLE) or ratio (NRE) evaluations, at the cost of upfront training and potential miscalibration.

All three methods use the same simulated training pairs but learn different targets under different losses, so their finite-sample behaviour differs. In our ERGM implementation, NPE provides the most reliable amortised posterior, NLE provides broadly comparable one-off inference at the cost of MCMC, and NRE did not yield well-calibrated posteriors or posterior predictives under the same simulation budget and classifier choice.

**Table 6:** Table of wall-clock times for training and posterior sampling for the Lazega friendship implementation; for NLE and NRE, the reported sampling times correspond to 8 chains being updated in a single batched operation on the device. If the same computation were carried out with truly serial chains, we would expect the wall-clock cost to be up to roughly eight times the values reported here.

Method	Train (s)	Build (s)	Inferring $10^5$ posteriors (s)
NPE	1154.23	0.03	0.18
NLE	760.87	0.24	4594.73 (8 vectorised chains)
NRE	613.57	0.01	1876.57 (8 vectorised chains)

## Discussion

Through our implementations of NPE and SNPE on ERGMs, we have established their appropriateness. We have shown that the resulting estimators can achieve adequate accuracy across a variety of network topological structures, in both the parameter space and the graph space (summary-statistics space). More importantly, NPE can achieve performance comparable to standard Bayesian ERGM fitting. This fulfils our main research aim of assessing the use of NPE for ERGMs. We also demonstrate the approach on a real-world network example and provide a detailed comparison with other closely related neural simulation-based approaches.

Furthermore, our study demonstrates the core advantage of amortised NPE for ERGMs, namely scalability. In Section 3.5, we systematically compare the fitting costs of NPE and standard Bayesian ERGM estimation and show efficiency gains once inference is required for as few as one to three networks, with increasing benefits in larger-sample analysis. This scalability arises from amortisation. Once a conditional density estimator is trained, posterior sampling for new observations is obtained by fast forward passes. By contrast, standard Bayesian ERGM estimation requires refitting via simulation-intensive MCMC for each network, and other neural approaches such as NLE and NRE still typically require MCMC at inference time, which can be computationally inefficient for large-scale implementations. This has significant implications for recent ERGM applications where the research interest is to infer on a population of networks rather than a single network (Lehmann et al. 2021).

While NPE and SNPE perform well in most ERGM cases, we also highlight several issues that can lead to challenges in practice, including multi-modality,“boundary effects”, and coverage issues. These ERGM-specific challenges provide guidance for diagnosing and interpreting NPE fitting difficulties in future ERGM implementations.

### Multi-modality

In the ERGM context, multi-modality refers to situations where a single parameter value can characterise (through the ERGM sampling distribution) two or more distinct regions of graph space. In practice, multi-modality affects the quality of simulated training data, and data-parameter pairs become sensitive to the simulation procedure when sampling under parameters that induce multiple modes.

When realising a network, we are essentially exploring the graph space to search for a domain in the graph space that matches the ERGM parameters. Under multi-modality, the chain may exhibit sharp “jumps”in the graph space and, more importantly, poor mixing between modes. In particular, we often observe the chain becoming trapped in a dense-network mode rather than exploring a sparse-network mode. As a result, simulations can be sensitive to the choice of initial network and the MCMC length. More specifically, when we initialise the chain at a dense network, it may remain in a dense mode for a long period and fail to explore sparse modes. In contrast, when we initialise at a sparse network, the chain may initially explore sparse modes, but with a sufficiently long run it may transition to a dense mode and then mix poorly across modes. Furthermore, the severity of multi-modality depends on the model specification, including the chosen combination of summary statistics.

This has two implications for implementing NPE on ERGMs. First, NPE inference quality and accuracy can be sensitive to simulation initialisation and MCMC length, so these choices should be standardised and aligned when comparing NPE with Bayesian ERGM fits. Second, the training dataset can be harder to learn when it contains many parameters that induce multi-modal behaviour, and this issue tends to be more severe when targeting very sparse graph spaces. Conditional density estimators may be challenged by such network-parameter pairs and can show degraded performance. The risk can be amplified in SNPE when using atomic loss, which may exhibit posterior leakage under poor proposal coverage. A potential mitigation is to use more expressive conditional density estimators.

### Boundary effect

Another issue we highlight is the“boundary effect”. We use “boundary effect”to refer to the fact that large regions of parameter space map to extreme network configurations (i.e., near-empty or near-full networks). In particular, once an ERGM parameter value is sufficiently extreme, further increases in its magnitude may not lead to meaningfully different networks, because the ERGM distribution has already concentrated most of its mass near the boundary of the graph space. For example, as the edges parameter becomes sufficiently large and positive, simulated networks become increasingly close to complete graphs, and further increases can yield little additional change in the realised summary statistics. This behaviour can become more sensitive and harder to diagnose when multiple summary statistics are included. This is related to the well-known degeneracy issue in ERGMs (Handcock et al. 2003; Snijders et al. 2006; Mele 2017; Van der Pol 2019), where small changes in parameters can cause abrupt and unstable shifts of the ERGM distribution towards extreme regions of the graph space.

The “boundary effect”can produce sharp transitions or plateau-like behaviour in the mapping from $\boldsymbol{\theta }$ to the induced distribution of summary statistics. In practice, such behaviour can be challenging for neural conditional density estimation. Highly expressive density estimators may overfit these transition and plateau regimes, allocating overly diffuse posterior mass along directions that are weakly identified by the summary statistics, which can mask more informative structure elsewhere in the parameter space. In our experiments, these difficulties become most severe when the proposal coverage is wide and includes substantial regions associated with boundary behaviour and multi-modality, which increase the risk of posterior leakage.

### Coverage

In our implementations, we find that coverage issues affect NPE and SNPE-based ERGM inference in two ways. First, an overly narrow proposal can yield a biased or truncated posterior approximation that still produces posterior predictive distributions close to the observed summary statistics. This occurs because dependencies among ERGM summary statistics can allow similar realised statistics to be produced by distinct parameter values. In such cases, it can mask the fact that the posterior is effectively truncated by the proposal support and that the “true” posterior region is not being explored. The resulting posterior predictive alignment is typically not“perfect”, but it can look sufficiently plausible to be misleading. Therefore, providing sufficiently wide coverage is important to avoid biased estimation.

Second, although we desire adequate coverage, excessively wide coverage can interact with“boundary effects”and multi-modality. If the proposal spans large regions of parameter space associated with extreme graph configurations or multi-modal behaviour, NPE and SNPE may encounter additional training and inference challenges.

### Limitations and future work

While we have carefully justified each implementation procedure in this study, we also acknowledge several unavoidable limitations and outline directions for future work.

Firstly, we highlight that using NPE is like throwing data-parameter sets into a“black box”. It is challenging to investigate the “true” root causes of implementation difficulties and leakage issues that we encountered during our explorations. We approach this by auditing data inputs, monitoring loss functions, and validating varying inference setups. Our summarised ERGM-specific issues rely on our attempts, experiences and understandings of ERGMs. Future implementations of NPE on ERGMs may focus on a robust approach to diagnosing implementation difficulties.

While our study has pointed out some potential resolutions to the above issues, we have not systematically established implementation recommendations and guidelines in this study, which is not a trivial undertaking. Reinforcement learning-based NPE may potentially be useful (Ward et al. 2022). Future studies could target these aspects accordingly.

Finally, we have not systematically studied how the choice of conditional density estimator, network architecture, and ERGM summary statistics affects NPE performance. In practice, we explored the effect of network architecture as part of our cost and sensitivity experiments and we experimented with variations of these components during development. We provide general guidance in Appendix C, but a comprehensive, controlled comparison across estimators, architectures, and model specifications remains beyond the scope of this study.

### Summary

Our study builds a solid foundation for using NPE for inference on ERGMs by exploring implementation issues and methods to assess potential bias. Our results demonstrate a promising direction for future research and highlight specific areas to be addressed going forward.

## Figures and Tables

**Algorithm 3:**
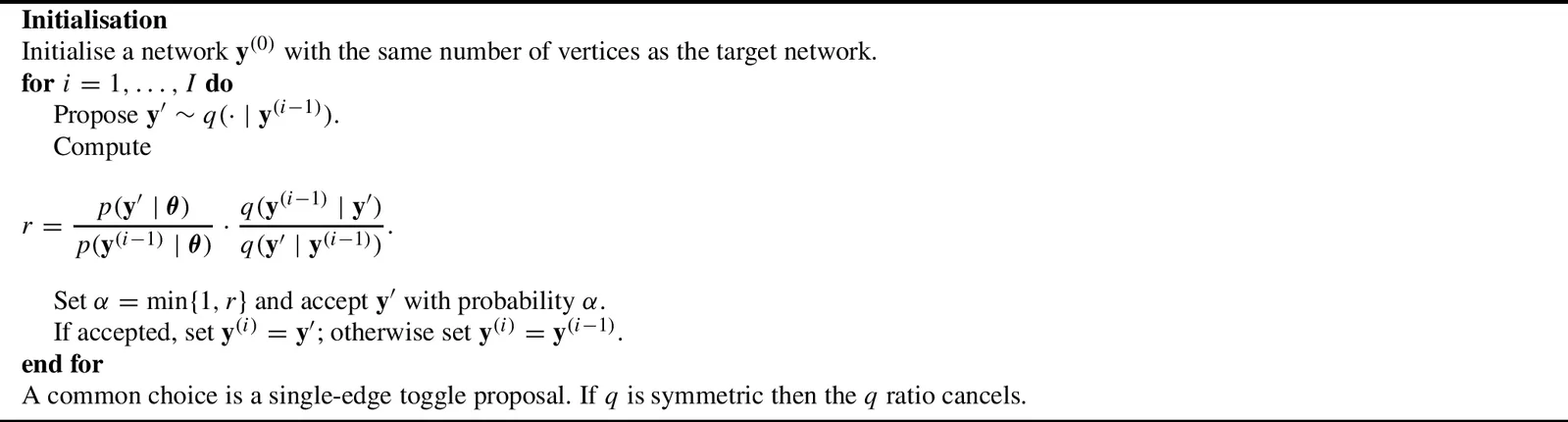
Network simulation (Metropolis-Hastings) given $\boldsymbol{\theta }$

## Data Availability

No datasets were generated or analysed during the current study.

## Appendix B sufficiency proof

Formally, we express the posterior distribution as:

$$\begin{aligned} \pi (\boldsymbol{\theta }\mid \textbf{y}) = \frac{\pi (\boldsymbol{\theta })}{p(\textbf{y})} \, \frac{\exp \!\bigl (\boldsymbol{\theta }^{\mathsf T}\textbf{h}(\textbf{y})\bigr )}{\sum _{\textbf{y}'\in \mathcal {Y}} \exp \!\bigl (\boldsymbol{\theta }^{\mathsf T}\textbf{h}(\textbf{y}')\bigr )}, \end{aligned}$$

and note that the expression depends on $\textbf{y}$ only through $\textbf{h}(\textbf{y})$. More specifically, the prior $\pi (\boldsymbol{\theta })$ is independent of $\textbf{y}$, and the sampling distribution $p(\textbf{y}\mid \boldsymbol{\theta })$ depends on $\textbf{y}$ only through $\textbf{h}(\textbf{y})$. The marginal likelihood

$$\begin{aligned} p(\textbf{y}) = \int \pi (\boldsymbol{\theta })\,p(\textbf{y}\mid \boldsymbol{\theta }) \,d\boldsymbol{\theta }, \end{aligned}$$

is defined in terms of the prior and sampling distributions, and therefore depends on $\textbf{y}$ only through $\textbf{h}(\textbf{y})$.

## Appendix C conditional density estimator

Our implementation relies on the Python package sbi (versions 0.22 and 0.24) (Tejero-Cantero et al. 2020). We discuss available conditional density estimators and provide high-level guidance on how to choose between them.

**Masked Autoregressive Flow (MAF)** is a normalising flow (Papamakarios et al. 2017). Intuitively, it “flows” probability mass through a sequence of invertible transformations. More specifically, let $z\sim p_Z$ be a base random variable and let x=f(z) be the transformed variable under an invertible and differentiable map *f*:

$$\begin{aligned} x=f(z). \end{aligned}$$

By the change-of-variables formula,

$$\begin{aligned} p_X(x)=p_Z\!\bigl (f^{-1}(x)\bigr )\left| \det \!\left( \frac{\partial f^{-1}(x)}{\partial x}\right) \right| , \end{aligned}$$

where $\det \!\left( \frac{\partial f^{-1}(x)}{\partial x}\right)$ is the Jacobian determinant of the inverse transformation.

MAF combines this flow construction with an autoregressive factorisation of a *D*-dimensional density,

$$\begin{aligned} p(x_1,\dots ,x_D)=\prod _{i=1}^{D} p(x_i\mid x_{1:i-1}), \end{aligned}$$

so that each component $x_i$ is modelled conditionally on the preceding components.

Concretely, MAF uses an affine autoregressive transformation

$$\begin{aligned} x_i=z_i\exp (\alpha _i)+\mu _i, \end{aligned}$$

where

$$\begin{aligned} \mu _i=f_{\mu _i}(x_{1:i-1}),\; \alpha _i=f_{\alpha _i}(x_{1:i-1}),\; z_i\sim \mathcal {N}(0,1), \end{aligned}$$

and $f_{\mu _i}$ and $f_{\alpha _i}$ are masked feed-forward neural networks. This implies

$$\begin{aligned} p(x_i\mid x_{1:i-1})=\mathcal {N}\!\bigl (\mu _i,\exp (2\alpha _i)\bigr ). \end{aligned}$$

In our study, we vary the number of hidden units to control the capacity of the flow.

Finally, we can compose multiple transformations of the same type, so the base distribution is mapped through a sequence of flow steps. We refer to the number of such steps as the number of transformations, which helps model multi-modal behaviour in ERGMs.

**Neural Spline Flow (NSF)** is also a normalising flow model. Instead of affine transformations, it uses spline-based transformations, typically monotonic rational quadratic splines (Durkan et al. 2019). It partitions the input domain [-B,B] into *K* intervals using K+1 knots. Let $$\{u_0,\dots ,u_K\}$$ denote the knot locations on the input axis and let $$\{v_0,\dots ,$$$$v_K\}$$ denote the corresponding knot values on the output axis. Suppose $$x\in [u_k,u_{k+1}]$$. We compute the local secant slope

$$\begin{aligned} s^{(k)}=\frac{v_{k+1}-v_k}{u_{k+1}-u_k}, \end{aligned}$$

and the normalised coordinate within the interval

$$\begin{aligned} \xi =\frac{x-u_k}{u_{k+1}-u_k}. \end{aligned}$$

The spline transformation in this interval is then

$$\begin{aligned} \begin{aligned} f(x)&= v_k+(v_{k+1}-v_k)\, \\&\quad \times \frac{ s^{(k)}\xi ^2+\delta _k\,\xi (1-\xi ) }{ s^{(k)}+\bigl (\delta _{k+1}+\delta _k-2s^{(k)}\bigr )\,\xi (1-\xi ) }. \end{aligned} \end{aligned}$$

where $\delta _k$ and $\delta _{k+1}$ denote the derivatives at the knots. Neural networks output the spline parameters (knot values and derivatives, subject to monotonicity constraints), and, as with MAF, NSF can stack multiple transformation layers.

**Mixture Density Network (MDN)** considers a mixture of Normal distributions with *P* components,

$$\begin{aligned} q_{\phi }(\theta \mid x) = \sum _{i=1}^{P} w_{i}(x)\, \mathcal {N}\!\left( \theta \mid \mu _{i}(x), \sigma _{i}^{2}(x)\right) . \end{aligned}$$

$w_{i}(x)$ is the mixing weight for component *i*, and the weights sum to 1. $\mu _{i}(x)$ and $\sigma _{i}(x)$ are the component means and standard deviations. A neural network (parameterised by ϕ) outputs $\{w_{i}(x), \mu _i(x), \sigma _i(x)\}_{i=1}^{P}$ from the input data *x*. The mixing weights $w_i(x)$ use a softmax activation function to ensure the weights sum to one, and the standard deviations $\sigma _i(x)$ use exponential or softplus activation functions. Put simply, an MDN uses a neural network to predict a mixture of Normal distributions to capture relationships between inputs and outputs.

In our study, we mainly use MAF with the default sbi package setup, which we consider sufficient for our purposes. We may adopt a deeper MAF (by increasing the number of transformations) to better capture complex ERGM densities, including sharp peaks and multi-modal structure. Alternatively, we may adjust the number of hidden units to control overall expressiveness and to avoid overfitting. As a general guideline, if a deeper MAF is still not expressive enough to model the target distribution, NSF can be considered instead, since its spline-based transformations can better capture complex non-linear relationships. By comparison, although MAF is also expressive, it is constrained by the linearity of its affine transformations. In addition, MDNs can be used to model multi-modal distributions via their mixture structure. MDNs also play an important role in NPE, especially for SNPE, because they allow use of the non-atomic loss. In sbi, this requires an MDN density estimator together with a compatible proposal and prior.

## Appendix D atomic proposal and leakage issue

While SNPE correctly targets the posterior, the normalising constant

$$\begin{aligned} Z_{\phi }(\textbf{x})=\int _{\boldsymbol{\theta }} q_{\phi }(\boldsymbol{\theta }\mid \textbf{x})\dfrac{\tilde{p}(\boldsymbol{\theta })}{\pi (\boldsymbol{\theta })} d\boldsymbol{\theta } \end{aligned}$$

is not always possible to evaluate. This depends on the choice of density estimator and proposal distribution. When the density estimator is an MDN and the proposal is Gaussian, SNPE can be used directly with a closed-form solution. Otherwise, SNPE uses an atomic loss so that we can flexibly use different density estimators and proposal distributions. In particular, the flow-based density estimator used in this study requires the atomic loss.

In the atomic loss, we“replace”integrals with sums. More specifically, we discretise the parameter space by considering finite sets (with mini-batch size *M*),

$$\begin{aligned} \boldsymbol{\theta } = \{\boldsymbol{\theta }_1, \boldsymbol{\theta }_2, \dots , \boldsymbol{\theta }_M\},\qquad \boldsymbol{\theta }_i \sim \tilde{p}(\boldsymbol{\theta }), \end{aligned}$$

where the atomic set $\boldsymbol{\theta }$ is a finite subset of parameters sampled from the“original”proposal (originally referred to as the hyper-proposal in (Greenberg et al. 2019)). We then define $U_{\boldsymbol{\theta }}(\cdot )$ as the uniform distribution over $\boldsymbol{\theta }$.

Under the atomic setting, the relationship between the proposal posterior and the actual posterior (Equation (4)) becomes

$$\begin{aligned} \tilde{q}_{\phi }(\boldsymbol{\theta }\mid \textbf{x}) = \frac{q_{\phi }(\boldsymbol{\theta }\mid \textbf{x}) / \pi (\boldsymbol{\theta })}{\sum _{\boldsymbol{\theta }' \in \boldsymbol{\theta }} \left( q_{\phi }(\boldsymbol{\theta }'\mid \textbf{x}) / \pi (\boldsymbol{\theta }') \right) }. \end{aligned}$$

Greenberg et al. (2019) showed that minimising the expected cross-entropy loss,

$$\begin{aligned} \mathcal {L}(\phi ) = - \mathbb {E}_{\boldsymbol{\theta }\sim \tilde{p},\, \boldsymbol{\vartheta }\sim U_{\boldsymbol{\theta }},\, \textbf{x}\sim p(\cdot \mid \boldsymbol{\vartheta })} \left[ \log \tilde{q}_{\textbf{x}, \phi }(\boldsymbol{\vartheta }) \right] , \end{aligned}$$

targets the correct proposal posterior and, consequently, the desired posterior.

In practice, $\boldsymbol{\theta }$ is sampled in mini-batches of size *M*, each representing an atomic set, so that we can use standard gradient-based optimisation. The atomic set is produced from all simulated data-parameter pairs, and, in SNPE, this includes pairs from earlier rounds. See Greenberg et al. (2019) for further details.

With the atomic loss, SNPE can be used flexibly with a range of density estimators, priors, and proposals. We also describe the leakage issue related to the atomic loss. Posterior leakage refers to the situation where the estimated posterior distribution assigns substantial probability mass to regions of the parameter space that should have little probability under the true posterior distribution. A range of issues can lead to leakage; here, we focus on reasons related to the prior and proposal distributions. SNPE using the atomic loss returns a distribution that is only proportional to the true posterior on the support of the proposal (or the prior, if starting from the prior). If the proposal is chosen to be excessively narrow, leakage can occur (Deistler et al. 2022). Therefore, adequate coverage of the posterior support is important.

## Appendix E other neural-based estimation methods

In addition to directly approximating the posterior $\pi (\boldsymbol{\theta }\mid \textbf{x})$, two other neural network-based approaches are commonly used in simulation-based inference. Although they are not our main focus, they are often considered comparable to NPE. We provide brief descriptions here. Further details can be found in the original publications.

**Neural likelihood estimation (NLE).** NLE trains a conditional neural density model $r_{\psi }$ (e.g., a MAF) to approximate the likelihood $p(\textbf{x}\mid \boldsymbol{\theta })$. Given simulated data-parameter pairs,

$$\begin{aligned} \boldsymbol{\theta }_{b} \sim \tilde{p}(\boldsymbol{\theta }), \qquad \textbf{x}_{b} \sim p(\textbf{x} \mid \boldsymbol{\theta }_{b}), \qquad b = 1,\dots ,B, \end{aligned}$$

NLE minimises the negative log-likelihood

$$\begin{aligned} \begin{aligned} \mathcal {L}_{\textrm{NLE}}(\psi )&= - \mathbb {E}_{\boldsymbol{\theta },\textbf{x}} \bigl [\log r_{\psi }(\textbf{x} \mid \boldsymbol{\theta })\bigr ] \\&\approx - \frac{1}{B} \sum _{b=1}^{B} \log r_{\psi }(\textbf{x}_{b} \mid \boldsymbol{\theta }_{b}). \end{aligned} \end{aligned}$$

The approximate posterior is then obtained via Bayes’ rule,

E1 $$\begin{aligned} \pi _{\textrm{NLE}}(\boldsymbol{\theta } \mid \textbf{x}) \;\propto \; r_{\psi }(\textbf{x} \mid \boldsymbol{\theta })\,\pi (\boldsymbol{\theta }), \end{aligned}$$

and standard Bayesian inference methods, such as Markov chain Monte Carlo, can be applied to (E1).

**Neural ratio estimation (NRE).** NRE targets the likelihood-to-marginal ratio $\rho (\boldsymbol{\theta },\textbf{x})$,

$$\begin{aligned} \rho (\boldsymbol{\theta },\textbf{x}) \;=\; \frac{p(\textbf{x} \mid \boldsymbol{\theta })}{m(\textbf{x})}, \qquad m(\textbf{x}) = \int p(\textbf{x} \mid \boldsymbol{\theta })\,\pi (\boldsymbol{\theta })\,\textrm{d}\boldsymbol{\theta }, \end{aligned}$$

by training a discriminative classifier $D_{\varphi }$ to distinguish samples from the joint distribution and the product of marginals on $(\boldsymbol{\theta },\textbf{x})$:

$$\begin{aligned} p_{\textrm{joint}}(\boldsymbol{\theta },\textbf{x}) = \pi (\boldsymbol{\theta })\,p(\textbf{x} \mid \boldsymbol{\theta }),\; p_{\textrm{prod}}(\boldsymbol{\theta },\textbf{x}) = \pi (\boldsymbol{\theta })\,m(\textbf{x}). \end{aligned}$$

With class label $z \in \{0,1\}$ indicating whether a pair is drawn from $p_{\textrm{joint}}$ (z=1) or $p_{\textrm{prod}}$ (z=0), the cross-entropy loss is

$$\begin{aligned} \begin{aligned} \mathcal {L}_{\textrm{NRE}}(\varphi )&= - \mathbb {E}_{\boldsymbol{\theta },\textbf{x},z} \biggl [ z \log D_{\varphi }(\boldsymbol{\theta },\textbf{x}) \\&\quad \quad + (1-z)\log \bigl (1 - D_{\varphi }(\boldsymbol{\theta },\textbf{x})\bigr ) \biggr ]. \end{aligned} \end{aligned}$$

At the optimum, the classifier satisfies

$$\begin{aligned} \frac{D_{\varphi ^\star }(\boldsymbol{\theta },\textbf{x})}{1 - D_{\varphi ^\star }(\boldsymbol{\theta },\textbf{x})} \;\approx \; \frac{p_{\textrm{joint}}(\boldsymbol{\theta },\textbf{x})}{p_{\textrm{prod}}(\boldsymbol{\theta },\textbf{x})} \;=\; \frac{p(\textbf{x}\mid \boldsymbol{\theta })}{m(\textbf{x})} \;=\; \rho (\boldsymbol{\theta },\textbf{x}). \end{aligned}$$

Since $m(\textbf{x})$ does not depend on $\boldsymbol{\theta }$, the posterior is proportional to

$$\begin{aligned}\pi _{\textrm{NRE}}(\boldsymbol{\theta } \mid \textbf{x}) \;\propto \; \rho _{\varphi ^{\star }}(\boldsymbol{\theta },\textbf{x})\,\pi (\boldsymbol{\theta }). \end{aligned}$$
